# *OeBAS* and *CYP716C67* catalyze the biosynthesis of health‐beneficial triterpenoids in olive (*Olea europaea*) fruits

**DOI:** 10.1111/nph.18863

**Published:** 2023-04-03

**Authors:** Fiammetta Alagna, James Reed, Ornella Calderini, Ramesha Thimmappa, Nicolò G. M. Cultrera, Alice Cattivelli, Davide Tagliazucchi, Soraya Mousavi, Roberto Mariotti, Anne Osbourn, Luciana Baldoni

**Affiliations:** ^1^ Department of Energy Technologies and Renewable Sources National Agency for New Technologies, Energy and Sustainable Economic Development (ENEA), Trisaia Research Centre 75026 Rotondella Italy; ^2^ Department of Biochemistry and Metabolism John Innes Centre Norwich Research Park Norwich NR4 7UH UK; ^3^ Institute of Biosciences and Bioresources National Research Council (CNR) 06128 Perugia Italy; ^4^ Amity Institute of Genome Engineering Amity University Uttar Pradesh Noida 201313 India; ^5^ Department of Life Sciences University of Modena and Reggio Emilia 42100 Reggio Emilia Italy

**Keywords:** bioactive compound, cytochrome P450, maslinic acid, oleanolic acid, olive oil, terpene, trait‐associated marker, β‐amyrin synthase

## Abstract

- The bioactive properties of olive (*Olea europaea*) fruits and olive oil are largely attributed to terpenoid compounds, including diverse triterpenoids such as oleanolic, maslinic and ursolic acids, erythrodiol, and uvaol. They have applications in the agri‐food, cosmetics, and pharmaceutical industries. Some key steps involved in the biosynthesis of these compounds are still unknown.
- Genome mining, biochemical analysis, and trait association studies have been used to identify major gene candidates controlling triterpenoid content of olive fruits.
- Here, we identify and functionally characterize an oxidosqualene cyclase (OeBAS) required for the production of the major triterpene scaffold β‐amyrin, the precursor of erythrodiol, oleanolic and maslinic acids, and a cytochrome P450 (CYP716C67) that mediates 2α oxidation of the oleanane‐ and ursane‐type triterpene scaffolds to produce maslinic and corosolic acids, respectively. To confirm the enzymatic functions of the entire pathway, we have reconstituted the olive biosynthetic pathway for oleanane‐ and ursane‐type triterpenoids in the heterologous host, *Nicotiana benthamiana*. Finally, we have identified genetic markers associated with oleanolic and maslinic acid fruit content on the chromosomes carrying the *OeBAS* and *CYP716C67* genes.
- Our results shed light on the biosynthesis of olive triterpenoids and provide new gene targets for germplasm screening and breeding for high triterpenoid content.

## Introduction

Olive (*Olea europaea*) is one of the most important oil fruit tree crops world‐wide. Its cultivation has been traditionally spread along the Mediterranean region, but is now expanding to other areas due to increased demand for olive oil for human consumption due to its health benefits (Baldoni & Belaj, 2010). Olive oil and fruits are the main source of fatty acids in the healthy Mediterranean Diet (UNESCO, 2013), and bioactive triterpenoids from olive have important pharmacological properties, such as anti‐inflammatory, antioxidant, anticancer, hepatoprotective, hypoglycemic, neuroprotective, and lipid‐lowering effects (Sánchez‐Quesada *et al*., 2013; Claro‐Cala *et al*., 2022; Papadaki & Tsimidou, 2022).

Triterpenoids such as oleanolic and maslinic acids, which are consumed as components of olive oil, are associated with improved endothelial function (de la Torre *et al*., 2020), while others such as oleanolic acid, maslinic acid, erythrodiol, and uvaol are considered responsible for cardiovascular risk protection (Rodriguez‐Rodriguez, 2015) and may also protect against neurodegenerative diseases (Qian *et al*., 2011). Many of these activities are associated with the ability of these compounds to modulate multiple signaling pathways such as the inflammatory and cell‐death pathways, and to stimulate the cellular antioxidant defense system (Reyes‐Zurita *et al*., 2009). For example, oleanolic and ursolic acids have been shown to increase the activity of antioxidative enzymes and to inhibit the cellular inflammatory process leading to promotion of expression of anti‐inflammatory cytokines (i.e. IL‐4 and IL‐10) and downregulation of the NF‐κB pathway and genes encoding proinflammatory proteins (Gudoityte *et al*., 2021). Moreover, oleanolic acid modulates signaling cascades and miRNA involved in oncogenesis, while ursolic acid is cytotoxic toward colorectal cancer cells, demonstrating significant potential in cancer prevention and therapy (Žiberna *et al*., 2017). Recent evidence has shown that betulinic, ursolic, and maslinic acids from olive may impair the viral replication of SARS‐CoV‐2, potentially leading to COVID‐19 treatment (Alhadrami *et al*., 2021).

The possibility of increasing triterpenoid content in olive fruits or oil is currently being investigated, in the light of the range of biological activities that olive triterpenoids offer. Different factors affect fruit and oil triterpenoid content, as the cultivar, the fruit ripening stage, and the oil extraction methods (Allouche *et al*., 2009, 2010; Papadaki & Tsimidou, 2022), the processing technologies for debittering table olives (Romero *et al*., 2010). Triterpenoids in the extra‐virgin olive oil (EVOO) reach up to 200 mg kg^−1^, representing the 4–6% of the fruit total content (Xie *et al*., 2021; Papadaki & Tsimidou, 2022).

High variability in triterpenoid content of olive fruits and olive oil appears to be strictly cultivar‐dependent, some having very low levels (Allouche *et al*., 2009; Lerma‐García *et al*., 2011; Lukić *et al*., 2013). A study including 40 olive varieties showed that the concentration of oleanolic and maslinic acids in the virgin olive oil ranged, respectively, from 3 to 79 mg kg^−1^ and from 4 to 50 mg kg^−1^ among different varieties (Allouche *et al*., 2009). Such variation, of *c*. 26‐fold for oleanolic acid and 12.5‐fold for maslinic acid, suggests that the selection of high triterpenoid cultivars may offer a potential route to increase the triterpenoid content of olive oil and, consequently, improving its beneficial effects on human health. This is supported by *in vivo* animal studies and human clinical trials that associated the health benefits of virgin and EVOO consumption with the presence of triterpenoids (Papadaki & Tsimidou, 2022). To assess whether the intake of oleanolic acid (OA) is effective in the prevention of metabolic syndrome or diabetes, OA‐enriched olive oils were elaborated by adding OA from olive leaves to refined olive oils (Santos‐Lozano *et al*., 2019; Fernández‐Aparicio *et al*., 2021).

Knowledge of target genes involved in triterpenoid biosynthesis and regulation is thus needed for germplasm screening and to develop genomics‐assisted breeding strategies.

**Fig. 1:**
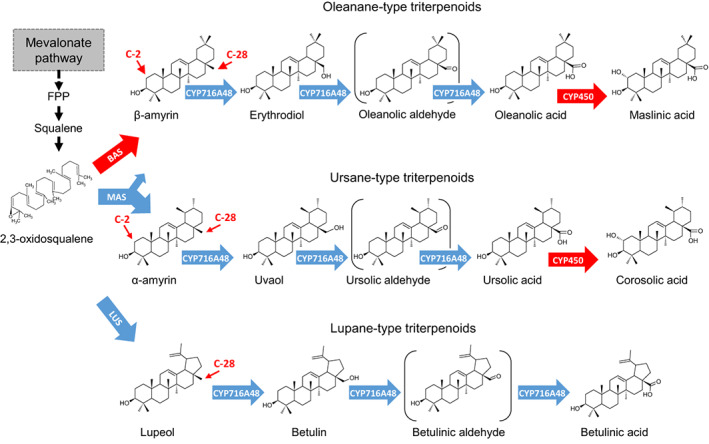
Olive triterpenoid biosynthesis. A schematic representation of the biosynthesis of olive main triterpenoids. The 2,3‐oxidosqualene originates from the mevalonic pathway. Red arrows indicate genes not yet characterized in olive and blue arrows indicate characterized steps in olive. The cyclization of 2,3‐oxidosqualene by oxidosqualene cyclases (OSCs) generates simple triterpene skeletons such as β‐amyrin, α‐amyrin, and lupeol. Two OSCs were currently characterized in olive: the multifunctional α‐amyrin synthase (OeMAS; Saimaru *et al*., 2007), which produces primarily α‐amyrin, and the lupeol synthase (OeLUS; Shibuya *et al*., 1999). The β‐amyrin synthase (OeBAS) is characterized in this work. The first oxidation reaction proceeds through three steps producing intermediates, which have a hydroxyl and aldehyde group at C‐28. These reactions are catalyzed by CYP716A48 (Suzuki *et al*., 2018), a CYP450 belonging to the 716A subfamily. The products are subjected to a following oxidation step not yet characterized in olive that is the object of this work. FPP, farnesyl diphosphate.

Oleanolic acid, maslinic acid, uvaol, ursolic acid, and erythrodiol are the major triterpenoids that accumulate in olive fruits and leaves and are also found in EVOO (Allouche *et al*., 2009; Stiti & Hartmann, 2012; Papadaki & Tsimidou, 2022). Olive is still the main source of commercial oleanolic acid (Jäger *et al*., 2009; Pollier & Goossens, 2012). The triterpenoids found in olive are derived from the simple triterpene scaffolds β‐amyrin, α‐amyrin, and lupeol. These scaffolds are formed by enzymes known as oxidosqualene cyclases (OSCs; Thimmappa *et al*., 2014). They then undergo a series of oxidations most likely mediated by cytochrome P450 monooxygenases (CYP450s) to give the structures shown in Fig. 1.

Despite the importance of olive triterpenoids, the main enzymes involved in their biosynthesis have not been yet characterized. Key missing pathway enzymes are the OSC involved in the formation of β‐amyrin (the precursor of the most abundant olive fruit triterpenoids, that is, erythrodiol, oleanolic, and maslinic acids), and the enzymes (most likely CYP450s) involved in the oxidation of oleanolic and ursolic acids to produce maslinic and corosolic acids, respectively (shown in red in Fig. 1). Only, a few genes involved in olive triterpenoid biosynthesis have been previously identified, such as those for the multifunctional OSC *OeMAS*, which produces primarily α‐amyrin (Saimaru *et al*., 2007), a lupeol synthase (*OeLUS*; Shibuya *et al*., 1999), and recently a CYP450 (*CYP716A48*) responsible for C‐28 oxidation of β‐amyrin, α‐amyrin, and lupeol (Suzuki *et al*., 2018).

Here, we clone and functionally characterize the genes encoding two enzymes that catalyze critical steps in biosynthesis of key olive triterpenoids, namely the β‐amyrin synthase BAS and the CYP450 required for 2α oxidation of the oleanane‐ and ursane‐type skeletons to produce maslinic acid and corosolic acid, respectively. In addition, through heterologous expression, we reconstitute the entire biosynthetic pathways for the synthesis of oleanane‐ and ursane‐type triterpenoids by transient expression in *Nicotiana benthamiana*. Furthermore, through trait association analysis we locate the major gene candidates controlling oleanolic and maslinic acid content in olive fruits, so providing the first insights into the chromosomal regions controlling triterpenoid content.

## Materials and Methods

### Identification of candidate genes

Olive (*Olea europaea* L.) OSCs were identified from olive genomic and transcriptomic data (Alagna *et al*., 2009; Jiménez‐Ruiz *et al*., 2020) by searching for genes annotated as squalene cyclases (InterPro Domain: IPR018333). In addition, basic local alignment (Blast) was used to search for sequences showing similarity to two previously characterized olive OSCs, the multifunctional α‐amyrin synthase *OeMAS* (GenBank accession no. AB291240.1; Saimaru *et al*., 2007), and the lupeol synthase (*OeLUS*; Shibuya *et al*., 1999). We also mined these datasets for candidate cytochromes P450. Genes with InterPro Domain: IPR001128 were selected as CYP450. Among them, genes annotated as CYP716, as assessed by Blast homology search with TAIR10, GenBank NR, and SwissProt (Jiménez‐Ruiz *et al*., 2020) were selected for further analyses.

Candidate genes putatively involved in the biosynthesis of fruit triterpenoid were selected based on phylogenetic and expression analyses. The candidate genes were amplified from fruit cDNA of cv Leccino and re‐sequenced. The closest homologs, namely *OeBAS CYP716A48v1* and *CYP716C67*, were selected for further analyses. For *OeBAS* and *CYP716C67*, two different alleles (*v1* and *v2*) per gene were cloned and characterized. The new CYP450s have been formally assigned names by the P450 naming committee.

### Phylogenetic analysis

Oxidosqualene cyclase and CYP450 amino acid sequences were aligned using ClustalW with default parameters, as implemented in the program MEGA10 (Kumar *et al*., 2018). Positions with gaps and missing data were eliminated. Evolutionary distances were computed using the JTT matrix‐based method (Jones *et al*., 1992). Phylogenetic analysis was carried out using a neighbor‐joining method with 1000 bootstrap replicates. CYP716 family members and their classification were retrieved from the cytochrome P450 homepage (Nelson, 2009) and recent papers.

### Co‐expression analyses

The expression values were retrieved by transcriptomic data of olive organs (Ramírez‐Tejero *et al*., 2020) and mapped to cv Picual genome (Jiménez‐Ruiz *et al*., 2020). Hierarchical clustering of putative OSCs and target genes characterized in this study was performed through MultiExperiment Viewer MeV v.4.9.0 (Howe *et al*., 2011). For each gene, log_2_ RPKM was used for clustering analysis. Pearson correlation was applied as distance metric and Average Linkage clustering as linkage method. Co‐expression of *CYP716s* with *OeBAS* was calculated by applying the Pearson correlation coefficient (Table S1).

### cDNA synthesis and RT‐qPCR analyses

A pool of different developmental stages has been collected for each organ (leaf, flower, fruit, and root) of cv Leccino plants from three biological replicates (olive clones). Immediately after harvest, samples were frozen in liquid nitrogen and stored at −80°C.

Total RNA from 200 mg of olive tissue was extracted with the RNeasy Plant Mini Kit (Qiagen) and treated with DNase I (Ambion, Thermo Fisher, Waltham, MA, USA). Reverse transcription of 2 μg of RNA was performed using oligo(dT)18 and the SuperScript III Reverse Transcriptase Kit (Invitrogen, Thermo Fisher), according to manufacturer's instructions. Reverse quantitative real‐time PCR was performed on a PCR Real‐Time 7300 (Applied Biosystems, Foster City, CA, USA), according to the manufacturer's protocol and using the Reagent kit for SYBR Green analysis (Thermo Fisher) and gene‐specific primers (Table S2). A standard curve was used to calculate primer efficiency. All reactions were performed in triplicate. After each assay, a dissociation kinetics analysis was performed to verify the specificity of the amplification products. Relative amounts of all mRNAs were calculated using the $2^{-\Delta \Delta C_{t}}$ method (Livak & Schmittgen, 2001), where Δ*C*_t_ = *C*_t_(target gene) − *C*_t_(reference gene). The housekeeping gene elongation factor (EF1α) was used as an endogenous reference for normalization. Statistically significant differences were determined by ANOVA, followed by Tuckey's HSD *post hoc* test on three biological replicates (**, *P* < 0.01; *n* = 3).

### Generation of constructs for heterologous expression

The entire coding sequences (CDSs) of *OeBAS*, *OeMAS*, *CYP716A48*, and *CYP716C67* were amplified from cDNA of olive leaves of cv Leccino, using iProof High‐Fidelity DNA Polymerase (Bio‐Rad) and gene‐specific primers (Table S2). *CYP72A67* was amplified from *Medicago truncatula* Gaertn. root cDNA using Phusion polymerase (NEB, Ipswich, MA, USA; Table S2). The *Centella asiatica* L. *CYP716C11* gene was synthesized with flanking Gateway® attB1 sites (IDT). *OeBAS*, *OeMAS*, and *CYP716A48* amplification products were cloned into the Gateway vector pCR8‐GWTOPO‐T/A (Thermo Fisher). *CYP716C67*, *CYP72A67*, and *CYP716C611* were cloned into pDONR207 using BP Clonase II Enzyme Mix (Thermo Fisher). All entry clones were sequenced to verify their integrity before LR reaction.

To generate constructs for yeast expression, *OeBAS* and *OeMAS* were subcloned into the vector pYES2‐DEST52, under the control of a galactose‐inducible promoter, using Gateway LR Clonase (Thermo Fisher), yielded the *Saccharomyces cerevisiae* expression constructs.

For the generation of constructs for *Nicotiana benthamiana* Domin. expression, *OeBAS*, *OeMAS*, *CYP716A48*, *CYP72A67*, and *CYP716C611* were subcloned from the relevant entry vectors (pCR8 or pDONR 207) into pEAQ‐*HT*‐DEST1 (Sainsbury *et al*., 2009) vector using LR Clonase Mix (Thermo Fisher).

### Yeast transformation

Yeast expression was carried out in the strain GIL77 (gal2 hem3‐6 erg7 ura3‐167; Kushiro *et al*., 2006). Yeast transformation was performed following standard protocols using PEG/LiAc (Yeastmaker™ Yeast Transformation System 2; Clontech Laboratories, Takara, Kusatsu, Japan). For functional analysis, yeast strains were grown at 28°C with shaking in 10 ml cultures in a selective medium (SD‐URA + 2% (w/v) glucose + supplements) until saturation (*c*. 2 d). The supplements were the following: 20 μg ml^−1^ ergosterol, 13 μg ml^−1^ hemin, and 13 μg ml^−1^ ampicillin. Cells were then pelleted, washed in ddH_2_O, transferred to an induction medium (SD‐URA + 2% (w/v) galactose + supplements), and incubated for a further day to allow accumulation of triterpenes. They were then pelleted before triterpene extraction.

### *Nicotiana benthamiana* agro‐infiltration

*Nicotiana benthamiana* plants were grown under glasshouse conditions, as described previously (Sainsbury *et al*., 2012). The pEAQ expression constructs were transformed into chemically competent *Agrobacterium tumefaciens* strain LBA4404 by flash freezing in liquid nitrogen. *Agrobacterium tumefaciens* strains carrying the target constructs were cultured and infiltrated by hand using a syringe, as described previously (Reed *et al*., 2017). Infiltrated leaves were harvested after 5 d and lyophilized.

### Triterpenes extraction and GC–MS analyses

Yeast pellet was suspended in 1 ml of saponification reagent (20% (w/v) KOH in 50% (v/v) ethanol) and incubated at 75°C for 1 h. Then, 0.5 ml of ddH_2_O was added and yeast culture was extracted twice with 1 ml of hexane. The resulting organic extracts were pooled and evaporated to dryness, and the residue was dissolved in 200 μl of hexane.

Ten milligram of *N. benthamiana* dried leaf material was weighed and pulverized with 2 mm tungsten beads (Qiagen) at 1000 rpm in a Geno/Grinder (Spex, Antylia Scientific, Vernon Hills, IL, USA). Five hundred microliters of ethyl acetate containing 50 μg ml^−1^ internal standard coprostanol (Sigma‐Merck, St Louis, MO, USA) was added to each tube, and samples were incubated at room temperature for 10 min with intermittent shaking. The samples were centrifuged at 13 000 ***g*** for 1 min, and 100 μl of the supernatant was transferred to a separate tube and dried under reduced pressure using a Genevac EZ‐2 Plus (SP Scientific, ATS, Warminster, PA, USA). The dried extracts were resuspended in 25 μl of derivatizing reagent TriSil‐Z (Sigma‐Merck) and transferred to a glass vial for GC–MS analysis. As comparison, triterpenes were also extracted by olive husks using the same procedure applied for *N. benthamiana* leaves.

### GC–MS analyses of yeast and plant extracts

GC–MS analyses were carried out on an Agilent 7890N GC system (Agilent technologies, Santa Clara, CA, USA) coupled to an Agilent 5977A mass selective detector in scan mode (*m*/*z* 60–800), with a solvent delay time of 8 min. All separations were performed with a ZB‐5HT column (30 m × 0.25 mm × 0.10 μm), using helium as carrier gas at 1 ml min^−1^ and with an injector temperature of 250°C. The program consisted of an initial temperature of 170°C for 2 min, then heated to 300°C at 20°C per min, and held at this temperature for further 11.5 min (total run time 20 min). Where available, mass spectra were compared with those of authentic standards including α‐amyrin, β‐amyrin, oleanolic acid, and ursolic acid (Extrasynthese, Genay, France). These standards were analyzed at a concentration of 250 μg ml^−1^.

### Genomic localization and gene cluster search

The CDSs of *OeMAS*, *OeBAS*, *OeLUS*, *CYP716A48*, and *CYP716C67* genes were used as query sequences to Blast search homologs in the olive genome of cv Leccino, v3 assembly (OLGENOME), and cv Arbequina (Rao *et al*., 2021). This information was used to localize genes on chromosomes and scaffolds (Table 1). Candidate genes for triterpenoid biosynthesis, positioned upstream in the triterpenoid biosynthetic pathway, were searched by Blast in the chromosomes of cv Leccino where markers linked to triterpenoid fruit content were identified (13 and 15), using candidate transcripts previously reported (Alagna *et al*., 2012). Presence of transcription factors (TFs) in the genomic regions surrounding the markers associated with oleanolic and maslinic acid content (from 1000 kbp before to 1000 kbp after the marker‐associated region) was investigated in the genome annotation of cv Leccino.

**Table 1:** Localization of target genes on olive genome.

Gene	cv Leccino			cv Arbequina		
	Chr	Position (kbp)	Gene ID	Chr	Position (kbp)	Gene ID
*OeBAS*	Chr 15	44 467	Oe15g534670.t01	Chr 15	376	GWHGAOPM030985
*OeMAS*	Chr 17	4625	Oe17g669990.t01	Chr 13	8713	GWHGAOPM027227
*OeLUS*	Chr 11	29 567	Oe11g122190.t01	Chr 17	8269	GWHGAOPM035713
*CYP716A48*	Chr 02	21 842	Oe2g436010.t01	Chr 02	24 884	GWHGAOPM003106
*CYP716C67*	Chr 13	36 989	Oe13g515570.t01	Chr 13	9881	GWHGAOPM027297

Position of target genes in olive genomes of cv Leccino, V3 assembly (OLGENOME), and cv Arbequina (Rao *et al*., 2021). Gene ID of best hits is reported, as assessed by Blast. Both v1 and v2 sequences of *OeBAS* and *CYP716C67* localize on the same genomic regions; thus, v1 and v2 are considered putative alleles.

Searches for biosynthetic gene clusters were performed using the plantiSMASH algorithm (Kautsar *et al*., 2017), by uploading olive genome sequences (cv Farga, OE9 assembly) and annotations (Cruz *et al*., 2016).

### Identification of markers linked to triterpenoid fruit content

#### Plant material

Olive fruits were collected from a full‐sib F1 progeny, represented by 94 15‐yr‐old trees, and from their parental varieties, Leccino and Dolce Agogia, field planted at CNR – Institute of Biosciences and Bioresources (Perugia, Italy). The harvest was carried out in early October, when most of fruits were at a ripening index of 3 (Furferi *et al*., 2010). Fruits were randomly chosen around the canopy and promptly frozen in liquid nitrogen and stored at −80°C.

#### Analysis of oleanolic and maslinic acid fruit content of Leccino × Dolce Agogia progeny

Triterpenes were extracted from olive fruits, following the protocol described previously (Mousavi *et al*., 2022), with minor modifications. Frozen olive fruits stored at −80°C (1.5 g) were mixed with 6 ml of dimethyl sulfoxide (DMSO) and homogenized with a mortar and pestle. The mixture was then sonicated for 5 min, vortexed for 1 min, and left for 24 h at 4°C. Thereafter, the mixture was further sonicated for 5 min, vortexed for 1 min, and centrifuged for 10 min at 4000 ***g***. The clear supernatant was filtered through a 0.22 μm PES (polyethersulfone) membrane filter and mixed with the internal standard (caffeic acid). The extraction was performed in triplicate. Identification and quantification of oleanolic and maslinic acids were carried out by liquid chromatography electrospray ionization ion trap mass spectrometry. The chromatographic conditions and the mass spectrometer parameters were fully described by Martini *et al*. (2018). Identification of compounds was carried out by comparing the retention times, the *m/z* values, and the fragmentation spectra (Figs S1, S2) with those of authentic standards in negative ionization mode. Quantification was performed by building calibration external curves with maslinic acid (from 10 to 100 mg l^−1^) and oleanolic acid (from 50 to 500 mg l^−1^) dissolved in DMSO (Fig. S3). The amount of maslinic and oleanolic acids was calculated by integrating the area under the peak measured from the extracted ion chromatograms (tolerance ±0.5 Da).

#### Marker–trait association analyses

For the Leccino × Dolce Agogia progeny, a genetic map was available, with a total of 16 743 segregating loci identified by ddRAD sequencing (Mariotti *et al*., 2020). Data on oleanolic and maslinic acids fruit content of the mapped cross progeny have been used to perform a linkage analysis with the segregating mapped loci, through the software package Tassel v.5 (Bradbury *et al*., 2007). To establish genotype/phenotype relationships, mixed linear model (MLM) was applied. To avoid possible spurious associations between molecular markers and genomic loci, the *Q* matrices were used as covariate in the association analysis. Marker position was calculated in an optimal size selection window of 100 base pairs, as reported previously (Peterson *et al*., 2012). Trait‐associated markers located in the same chromosomes of candidate genes have been selected.

## Results

### Identification of oxidosqualene cyclases involved in olive triterpenoid biosynthesis

**Fig. 2:**
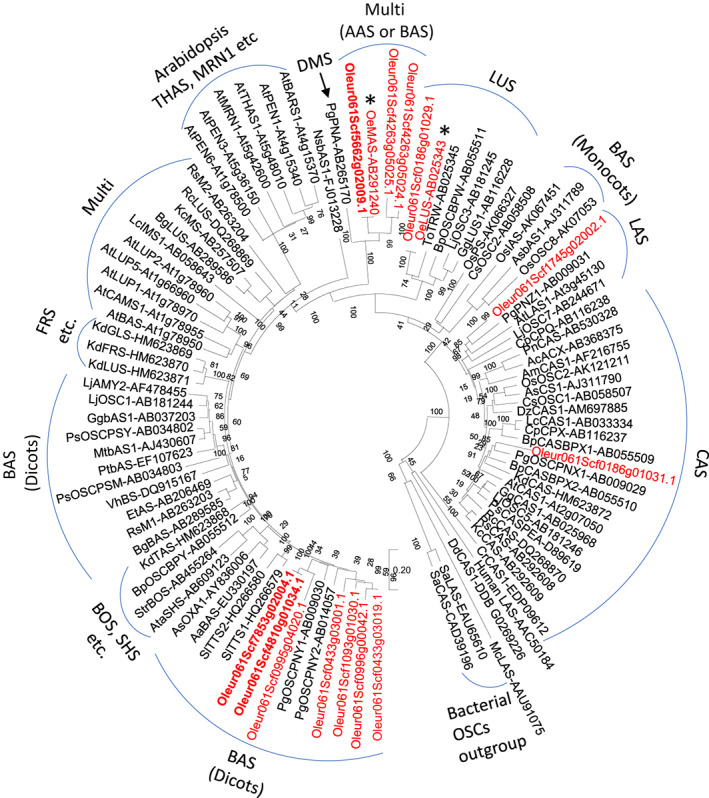
Phylogenetic analysis of olive oxidosqualene cyclases (OSCs). Neighbor‐joining (Nj) tree of functionally characterized OSCs from diverse taxa along with olive OSC candidates (shown in red). Olive oxidosqualene cyclases in red and bold were functionally characterized in the present study. Asterisk indicates the lupeol synthase OeLUS (Shibuya *et al*., 1999), and the multifunctional amyrin synthase OeMAS (Saimaru *et al*., 2007) previously characterized. Candidates were retrieved from olive transcriptomic data (Jiménez‐Ruiz *et al*., 2020). Different OSC clades are marked by blue arcs. Functionally characterized OSCs were given along with their GenBank accession numbers. Nj tree was drawn using Mega 10.2.6 as using parameters as shown in the methods. Bootstrap values are indicated at the branch nodes. AAS, α‐amyrin synthase; BAS, β‐amyrin synthase; BOS, baccharis oxide synthase; CAS, cycloartenol synthase; DMS, dammarenediol‐II synthase; FRS, friedelin synthase; LAS, lanosterol synthase; LUS, lupeol synthase; MRN, marnerol synthase; Multi, multifunctional OSCs; SHS, shionone synthase; THAS, thalianol synthase.

Systematic mining of the available olive genome and transcriptome sequence data (Alagna *et al*., 2009; Jiménez‐Ruiz *et al*., 2020; Ramírez‐Tejero *et al*., 2020) revealed 13 predicted OSCs. Phylogenetic analysis of the predicted protein sequences of these showed that seven grouped with previously characterized β‐amyrin synthases (BAS) from other plant species, while the remaining six were located in other clades (Fig. 2). Of these six, three grouped with multifunctional OSCs, including the previously characterized olive OSC OeMAS, which synthesizes α‐amyrin as its major product, along with smaller amounts of β‐amyrin, taraxasterol, and butyrospermol (Saimaru *et al*., 2007; Fig. 2). One of the hits clustered with the lupeol synthases subclade (LUS), which includes the previously characterized olive lupeol synthase OeLUS (Shibuya *et al*., 1999). The remaining two predicted olive OSCs were located in the sterol synthase subgroups, cycloartenol synthases (CAS) and lanosterol synthases (LAS). Analysis of the gene expression profiles of the 12 olive OSCs across different tissues (fruits, flowers, leaves, roots, and stems) revealed that two of these OSCs (both belonging to the dicot β‐amyrin synthases (BAS) subgroup shown in Fig. 2) were expressed at high levels in the fruits, consistent with a potential role in the biosynthesis of β‐amyrin‐derived triterpenoids in this organ. By contrast, the previously characterized mixed function OSC OeMAS showed very little expression in the fruits. For one OSC, expression data were not available, indicating that, presumably, it is not expressed in the selected samples.

**Fig. 3:**
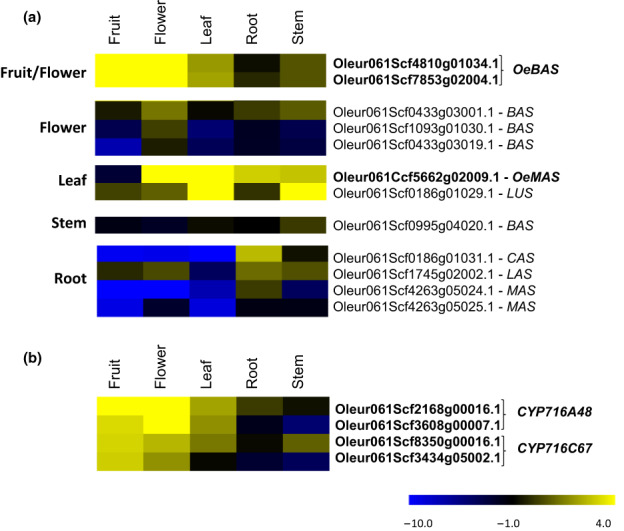
Heat map of olive oxidosqualene cyclases (OSCs) and selected CYP450s. (a) Heat map of putative OSCs. Genes were tentatively named based on their putative function as indicated by phylogenetic tree of Fig. 3 (BAS, β‐amyrin synthase; CAS, cycloartenol synthase; LAS, lanosterol synthase; LUS, lupeol synthase; MAS, multifunctional α‐amyrin synthase). (b) Heat map of candidate CYP450s involved in oleanolic and maslinic acid biosynthesis. For each gene, log_2_ RPKM was used for clustering analysis. The expression values were retrieved by Ramírez‐Tejero *et al*. (2020) and refer to transcripts mapped to Picual genome (Jiménez‐Ruiz *et al*., 2020). Transcripts in bold correspond to those functionally characterized in this study. OeMAS correspond to OEA; the multifunctional α‐amyrin synthase previously characterized (Saimaru *et al*., 2007). CYP716A48 was characterized by Suzuki *et al*. (2018). Two transcripts highly similar to each other were found for *OeBAS*, *CYP716A48*, and *CYP716C67*.

With the aim of characterizing the key missing steps in the biosynthesis of olive triterpenoids, we first focused on the candidate OSCs likely to be involved in the biosynthesis of β‐amyrin in the fruits. Among the BAS candidates, two (*Oleur061Scf4810g01034.1* and *Oleur061Scf7853g02004.1*) that had strong expression in the fruits (Fig. 3a) were selected for functional characterization. The CDSs of these two genes were amplified from cv Leccino. The predicted products of these genes share 99.8% amino acid sequence identity. The two genes were therefore namely β‐amyrin synthase *v1* and *v2* (*OeBASv1*, *v2*). Reverse transcribed quantitative PCR (RT‐qPCR) confirmed that *OeBAS* was highly expressed in fruits. By contrast, *OeMAS* showed very little expression in the fruits but was expressed strongly in other olive organs (Fig. S4).

### Functional characterization of *OeBAS* in yeast

**Fig. 4:**
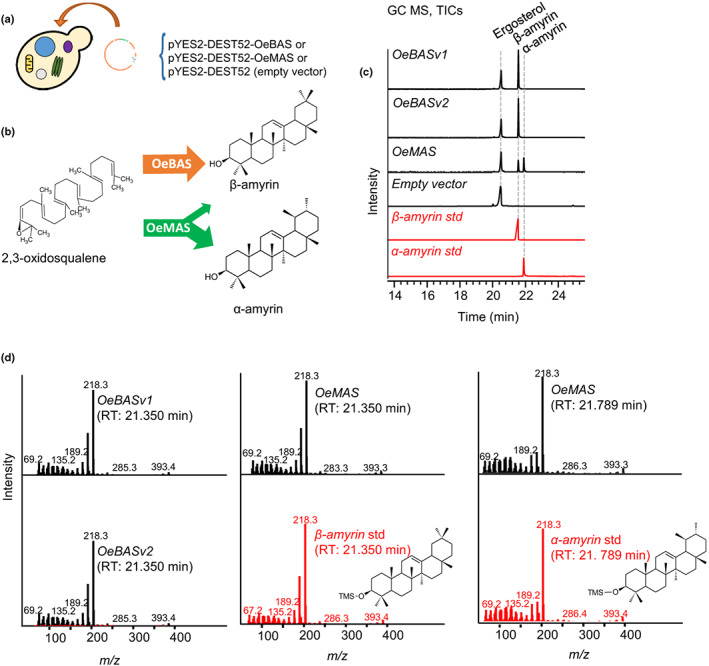
Functional characterization of *OeBAS* and *OeMAS* in yeast. (a) Yeast (*Saccharomyces cerevisiae*, GIL77 strain) transformation with target constructs. (b) Reactions catalyzed by OeBAS and OeMAS oxidosqualene cyclases. (c) GC–MS total ion chromatograms (TICs) of extracts from yeast cultures expressing *OeBAS*, *OeMAS*, or empty vector. (d) GC–MS mass spectra of the extracts. Data were compared with α‐amyrin and β‐amyrin standards (red indicated).

The full‐length CDSs of *OeBAS* (*v1* and *v2*) were cloned into the pYES2‐DEST52 expression vector under the control of a galactose‐inducible promoter and expressed in the yeast strain GIL77 (Kushiro *et al*., 2006). The previously characterized multifunctional olive OSC *OeMAS* was included as a control (Saimaru *et al*., 2007). Following galactose induction, cell extracts were analyzed by GC–MS. GC–MS analyses revealed that OeBAS produces β‐amyrin *in vivo* in yeast (Fig. 4). OeBASv1 and OeBASv2 yielded products with the same retention time and mass spectra. Due to diploid heterozygous nature of the olive genome (Rao *et al*., 2021), these are presumably two allelic forms of the same gene. OeBASv1 was selected for further experiments and hereafter it is referred to as OeBAS. In contrast to OeBAS, which produced only β‐amyrin, OeMAS produced both α‐ and β‐amyrin (Figs 4, S5), as reported previously (Saimaru *et al*., 2007).

### Identification of olive cytochrome P450s involved in oleanolic and maslinic acid biosynthesis

Oxidation of triterpene scaffolds is typically carried out by CYPs. The enzyme responsible for the C‐2 oxidation of triterpene scaffolds to produce maslinic and corosolic acids has not yet been identified in olive (Fig. 1). Members of the CYP716 family of cytochrome P450s are major contributors to triterpenoid scaffold oxidation and diversification in plants (Miettinen *et al*., 2017). The CYP716C11 has been shown to be responsible for the C‐2 oxidation of oleanolic to maslinic acid *C. asiatica* (Miettinen *et al*., 2017). Furthermore, CYP716 (CYP716A48v2) has previously been shown to be involved in the C‐28 oxidation of triterpene scaffolds in olive (Suzuki *et al*., 2018). We, therefore, prioritized members of the CYP716 family as potential candidates for the missing C‐2 oxidation step.

*CYP716* predicted CDSs were retrieved from an olive genomic dataset (Jiménez‐Ruiz *et al*., 2020; Ramírez‐Tejero *et al*., 2020). Out of 713 predicted CYP450 recovered, 19 CDSs encoding for putative *CYP716* were found. A phylogenetic tree of their predicted amino acid sequences was constructed (Fig. S6). This included sequences from representative members of different CYP716 subfamilies according to the cytochrome P450 homepage (Nelson, 2009) classification. The analysis indicated that the selected candidates effectively belong to CYP716 family. Some of the candidate CYPs have a very high amino acid identity and may correspond to isoforms of the same gene. This analysis showed that Oleur061Scf8350g00016.1 and Oleur061Scf3434g05002.1 (sharing 99% amino acid identity) grouped within the C subfamily together with the *C. asiatica* C‐2 oxidase CYP716C11 (GenBank AOG74835.1; Miettinen *et al*., 2017).

Hierarchical clustering analysis of RNA‐seq expression data of olive *OSCs* and *CYP716s* allowed us to identify sets of co‐expressed genes (Fig. S7). In order to identify the candidate CYP responsible for the triterpenoid production in olive fruits, we specifically searched for *CYP716s* co‐expressed with *OeBAS* (*Oleur061Scf4810g01034.1* and *Oleur061Scf7853g02004.1*). This analysis identified four *CYP716s* expressed in olive fruit (Fig. 3b) that were significantly co‐expressed with *OeBAS* (Pearson correlation coefficient > 0.7; Table S1; Fig. S7). Two of them (*Oleur061Scf2168g00016.1* and *Oleur061Scf3608g00007.1*) correspond to the *CYP716A48* previously characterized; the other two (*Oleur061Scf8350g00016.1* and *Oleur061Scf3434g05002.1*) are the two CYP716s belonging to C subfamily, identified by the phylogenetic analysis. These latter transcripts were tentatively named *CYP716C67*. These were selected for functional characterization together with the previously characterized *CYP716A48* (Suzuki *et al*., 2018).

The target genes were amplified from cv Leccino cDNA, cloned, and re‐sequenced. This allowed us to identify two sequences (*v1* and *v2* with 99.58% of identity) of *CYP716C67*. The amino acid sequence of the cloned CYP716A48v1 from cv Leccino differs by three amino acids from the previously characterized sequence (CYP716A48v2) reported from cv Nevadillo Blanco (Fig. S8). Further RT‐qPCR gene expression analysis of *CYP716A48* and *CYP716C67* in different organs suggested that the patterns were overall comparable with that of the RNA‐seq expression data. Some minor differences were observed, most likely due to differences in the varieties, developmental stage, and/or sampling conditions (Fig. S4). However, the analysis clearly confirmed that both *CYP716C67* and *OeBAS* are highly expressed in the fruits, consistent with a role in triterpenoid biosynthesis in olive fruits.

### Expression of the olive oleane‐ and ursane‐type triterpene pathway genes in *N. benthamiana*

Functional characterization of OSC and CYP candidates was carried out by transient expression in *N. benthamiana*. The target genes were expressed in combination with agro‐infiltration. A feedback‐insensitive version of the upstream mevalonate pathway gene *3‐hydroxy,3‐methylglutaryl‐CoA reductase* (*tHMGR*) was included to boost the supply of the triterpene precursor 2,3‐oxidosqualene (Reed *et al*., 2017). The candidates *OeBAS* and *CYP716C67* (v1 and v2), *CYP716A48v1*, and the previously characterized *OeMAS* genes were cloned into the pEAQ‐*HT*‐DEST1 expression vector (Sainsbury *et al*., 2009).

**Fig. 5:**
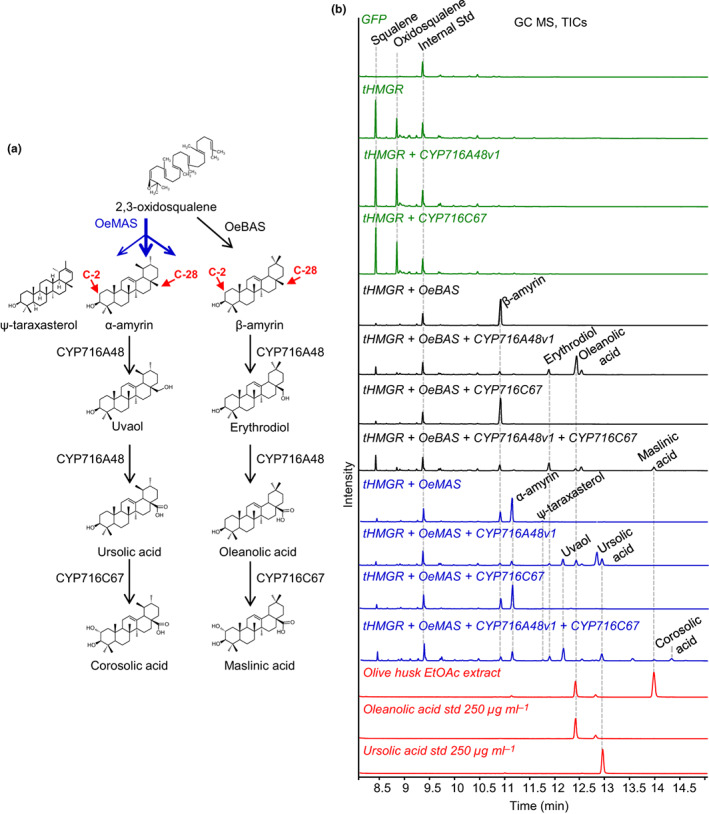
Expression of the olive biosynthetic pathway for oleanane‐ and ursane‐type triterpenoids in *Nicotiana benthamiana*. (a) The biosynthetic steps catalyzed by target genes. (b) GC–MS total ion chromatograms (TICs) of leaf extracts from *N. benthamiana* infiltrated with target constructs. Control reactions (green) of GFP‐expressing plants are shown at the top, followed by a *tHMGR*‐only control; the expression of *tHMGR* in the absence of an oxidosqualene cyclase results in the production of squalene and 2,3‐oxidosqualene; expression of *CYP716A48v1* or *CYP716C67* with *tHMGR* and no olive oxidosqualene cyclase (OSC) did not show any difference compared with the *tHMGR* controls. Expression of *OeBAS* (black) with *tHMGR*, *CYP716A48v1*, and *CYP716C67* results in the production of oleanane‐type triterpenoids. Expression of *OeMAS* (blue) with *tHMGR*, *CYP716A48v1*, and *CYP716C67* results in the production of both oleanane‐ and ursane‐type triterpenoids; TICs were compared with olive husk ethyl acetate extract, 250 μg ml^−1^ of oleanolic acid and ursolic acid standards (red). The retention times and mass spectra of the products generated by CYP716C67v1 and CYP716C67v2 were identical; thus, only the analyses performed by using CYP716C67v1 are reported.

Analysis of plant extracts by GC–MS confirmed the functions of *OeBAS* and *OeMAS*, as being consistent with those determined by expression in yeast (Figs 5, S9). An additional small peak was also detected in the leaf extracts of *OeMAS*‐expressing plants. The mass spectrum of this compound was found to be consistent with ψ‐taraxasterol (Lodeiro *et al*., 2007; Fig. S10). Co‐expression of *OeBAS* with *CYP716A48v1* resulted in two products. Based on retention times and mass spectra, the major product was identified as oleanolic acid and the minor product as erythrodiol (Figs 5, S11). Co‐expression of *OeBAS* with both *CYP716A48v1* and *CYP716C67v1/v2* resulted in the production of a new compound that, based on the retention time and mass spectra, was identified as 2‐α‐oleanolic acid (maslinic acid; Figs 5, S11). The retention times and mass spectra of the products generated by CYP716C67v1 and CYP716C67v2 were identical. Therefore, these two enzymes are referred as CYP716C67.

Co‐expression of *OeMAS* with *CYP716A48v1* resulted in the production of oleanolic and ursolic acids and their intermediates erythrodiol and uvaol, while *OeMAS*, together with both *CYP716A48v1* and *CYP716C67*, produced two additional compounds, assigned as 2‐α‐oleanolic acid (maslinic acid) and 2‐α‐ursolic acid (corosolic acid; Figs 5, S12). Co‐expression of either *BAS* or *MAS* with *CYP716C67* did not result in any additional peak (Fig. 5), indicating that CYP716C67 is able to oxidase both oleanolic and ursolic acids but is unable to use α‐ or β‐amyrin as substrates.

**Fig. 6:**
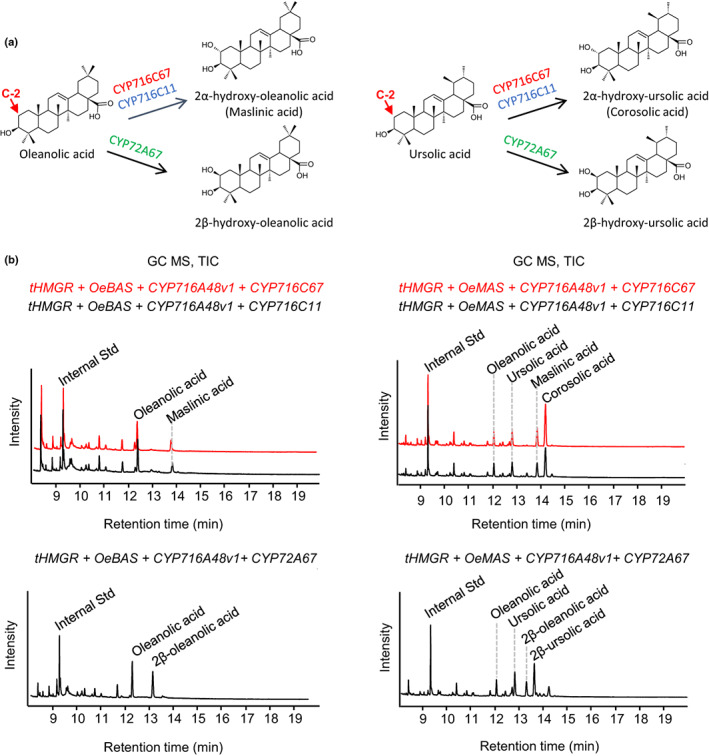
Combinatorial biosynthesis of oleanane‐ and ursane‐type triterpenoids in *Nicotiana benthamiana*. (a) Reactions catalyzed by different cytochromes P450: CYP716C67 from *Olea europaea*, oleanolic acid 2α hydroxylase from *Centella asiatica* (CYP716C11), oleanolic acid 2β hydroxylase from *Medicago truncatula* (CYP72A67). (b) GC–MS total ion chromatograms (TICs) of extracts from *N. benthamiana* leaves expressing *OeBAS* or *OeMAS* with *tHMGR* (Reed *et al*., 2017), *CYP716A48v1* and the different cytochromes P450. The retention times and mass spectra of the products generated by CYP716C67v1 and CYP716C67v2 were identical; thus, only the analyses performed by using CYP716C67v1 are reported.

In addition, we used a combinatorial biosynthesis approach to further clarify the functions of target genes. For comparison, *OeBAS* and *CYP716A48v1* were co‐expressed with either *C. asiatica CYP716C11* (oleanolic acid 2α hydroxylase, GenBank accession no. KU878852) or *M. truncatula CYP72A67* (oleanolic acid 2β hydroxylase, GenBank accession no. AB558149). Similarly, *OeMAS* was co‐expressed with the same genes. The retention time and mass spectra of the olive CYP716C67 products were an exact match to those of the *C. asiatica* CYP716C11, confirming that these CYP450s are 2α‐oxidases (Figs 6, S13). The mass spectrum of the *M. truncatula* CYP72A67‐derived compounds 2β‐hydroxyoleanolic acid and 2β‐hydroxyursolic acid are nearly identical to their 2α‐counterparts, the difference in retention time resulting from the different stereochemistry of the C‐2 hydroxyl group (Fig. S13).

GC–MS analysis confirmed that CYP716A48v1 is a C‐28 oxidase with activity toward the triterpene skeletons α‐amyrin and β‐amyrin. It produces, through three steps, oleanolic acid or ursolic acid, respectively, when β‐amyrin or α‐amyrin is used as substrate. *CYP716C67* encodes a 2α‐oxidase that is able to catalyze the downstream steps for the formation of either maslinic acid or corosolic acid, respectively when oleanolic acid or ursolic acid is provided as substrate. In summary, our identification of the enzymes involved in the biosynthesis of β‐amyrin and in the C‐2 oxidation of the triterpenoid skeleton completes the characterization of the main enzymes involved in the triterpenoid biosynthesis of olive fruits.

### Genomic localization and identification of gene clusters

Several triterpene pathways are encoded by biosynthetic gene clusters in plant genomes, whereby these genes are physically linked and co‐expressed (Thimmappa *et al*., 2014). Investigation of the chromosomal locations of the OSCs and CYP716s genes in the olive genome sequence assembly of cvs Leccino (OLGENOME) and Arbequina (Rao *et al*., 2021; Table S3) showed that *OeMAS*, *OeBAS*, *CYP716A48*, and *CYP716C67* are not physically clustered in a single genomic region but are for the most part located on different chromosomes (Table 1). Of note, however, *OeMAS* and *CYP716C67* are located on the same chromosome (chromosome 13) in cv Arbequina.

Analysis of olive genomic data (Cruz *et al*., 2016) using plantiSMASH, an algorithm that predicts potential biosynthetic gene clusters in plant genomes (Kautsar *et al*., 2017), confirmed that the characterized genes do not cluster in the olive genome. A putative biosynthetic gene cluster that includes the lupeol synthase gene *OeLUS*, together with three putative cytochrome P450 genes and a putative cycloartenol synthase gene was identified (Fig. S14). However, these genes did not appear to be co‐expressed in the selected organs. An additional 11 putative terpene clusters consisting of genes for predicted terpene synthases and potential tailoring enzymes (e.g. epimerases, glycosyltransferases, dioxygenases, CYP450s, aminotransferases, and acyltransferases), were also identified (Table S4). However, except for *OeLUS*, these clusters do not include OSC genes, indicating that presumably they are not involved in triterpenoid biosynthesis but may have roles in the biosynthesis of other types of terpenes.

### Identification of markers linked to oleanolic and maslinic acid fruit content

**Fig. 7:**
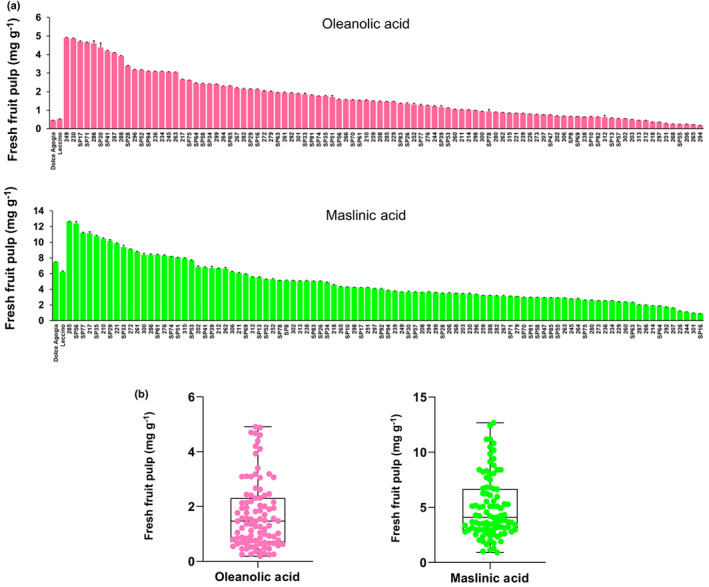
Variation of oleanolic and maslinic acid content in 96 individuals of the olive cross‐population Leccino × Dolce Agogia. (a) Histograms showing oleanolic and maslinic acid content of olive fruits. Bars represent means ± SD of three replicates. (b) Box and whiskers plots representing the distribution of the trait. These plots display the five‐number summary of a set of data: the minimum and maximum, the first quartile, the median, and the third quartile, allowing a comprehensive data comparison. Mann–Whitney test results show significant differences (*P* < 0.05) among phenotypic distribution. The circles represent the 96 individuals. Content is expressed as mg g^−1^ of fresh fruit pulp.

Clear segregation of the oleanolic and maslinic acid content was observed in the fruits of the crossing population Leccino × Dolce Agogia (Fig. 7). Oleanolic acid content varied from 0.18 to 4.9 mg g^−1^ of fresh fruit pulp, and maslinic acid from 0.91 to 12.66 mg g^−1^. The observed high variability highlights the importance of identifying the genes responsible for fruit triterpenoid content and represents the premise for the identification by genetic mapping of the genomic regions controlling the content in these compounds.

Marker–trait association analysis was performed to evaluate the association between target genes implicated in triterpene biosynthesis and oleanolic or maslinic acid content in fruits. Data for the oleanolic and maslinic acid content of the crossing population were merged with the segregating single‐nucleotide polymorphism (SNP) data available for the genetic map of that progeny (Mariotti *et al*., 2020). The genotype/phenotype relationships were estimated using the mixed lineage model (MLM) method. The list of the significant markers identified in the olive genome is reported in Table S5. The markers with *P* < 0.05 identified in the linkage groups bringing the candidate genes of triterpene biosynthesis (reported in Table 1) were selected.

**Fig. 8:**
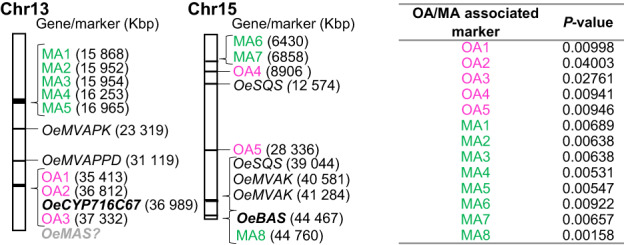
Markers significantly associated with oleanolic and maslinic acids content of olive fruits. Segregant restriction‐associated DNA (RAD) markers significantly associated with oleanolic (OA) and maslinic (MA) acids content were identified by using Leccino × Dolce Agogia map (Mariotti *et al*., 2020). Marker–trait association was performed with linear mixed‐effects model (MLM) incorporating structure *Q*‐matrix by using Tassel package. Markers localized on chromosomes 13 and 15 of cv Leccino genome (v3 assembly of OLGENOME Project) are shown. *P*‐values indicating significant genotype/phenotype association (*P* < 0.05) are indicated in the right table. The marker position is calculated in a size selection window of 100 base pairs (Peterson *et al*., 2012). Genes in bold were functionally characterized in the present study. *OeMVAK*, putative mevalonate kinase; *OeMVAPK*, putative phosphomevalonate kinase; *OeMVAPPD*, putative mevalonate diphosphate; *OeSQS*, putative squalene synthase.

This analysis identified 13 markers that were significantly associated with oleanolic and maslinic acid content (*P* < 0.05). These were located on chromosomes 15 and 13, where *OeBAS* and *CYP716C67* are respectively located (Fig. 8). The markers closest to the target genes are OA2 OA3 and MA8. OA2 and OA3 are significantly associated with oleanolic acid content and occur in chromosome 13, respectively, 173 and 343 kbp of distance from *CYP716C67*. MA8 is significantly associated with maslinic acid content and occurs in chromosome 15, 293 kbp of distance from *OeBAS*.

Further investigation of chromosomes 13 and 15 revealed the presence of other candidate genes positioned upstream in the triterpenoid biosynthetic pathway (Table S6). For example, in addition to harboring *OeBAS*, chromosome 15 of cv Leccino also contains putative mevalonate kinase (*OeMVAK*) and squalene synthase (*OeSQS*) genes. Also, chromosome 13, in addition to *CYP716C67*, has two genes of the mevalonate pathway, namely a putative phosphomevalonate kinase (*MVAPK*) and a mevalonate diphosphate decarboxylase gene (*MVAPPD*). These mevalonate pathway enzymes are involved in the biosynthesis of the triterpene precursor 2,3 oxidosqualene, the substrate for the OSCs that make the scaffolds of the triterpenoids found in olive fruit. Transcription factors in the genomic regions surrounding the molecular markers of chromosomes 13 and 15 have been also found (Table S7). Among them, TF belonging to bHLH and AP2‐like, and WRKY families have been identified. The TFs closest to the target genes are two *AP2‐like*, and a putative *bHLH52* located on chromosome 13, respectively, 103, 105, and 110 kbp of distance from *CYP716C67*, and a *bHLH57* located on chromosome 15, 192 kbp of distance from *OeBAS*.

These are the first data on the localization of olive triterpene genes in the olive genome. Despite further analyses are needed to verify the observed marker–trait associations, our data indicate that chromosomes 13 and 15, which bring markers associated with oleanolic and maslinic acid, as well as, diverse candidate genes involved in olive triterpenoid biosynthesis, are potential targets for the marker‐assisted selection of high triterpenoid genotypes within olive breeding programs.

## Discussion

The heightened interest of consumers in the nutritional quality of EVOO has highlighted the need to identify strategies to improve the profile of health‐promoting bioactive compounds in olive fruit (Pérez *et al*., 2018; Mousavi *et al*., 2022).

With the present work, we have identified and functionally characterized key enzymes required for the biosynthesis of olive triterpenoids that determine the quality of olive oil and have reconstituted the cognate pathways in *N. benthamiana*. We have further mapped the genes for biosynthesis of the main triterpenoids of olive fruits on the olive genome and identified SNP markers associated with oleanolic and maslinic acid content. This information can be used for the marker‐assisted selection of high triterpenoid genotypes within olive breeding programs.

A total of 13 predicted OSC and 19 CYP716 genes were identified by mining olive transcriptomic data. Among these, *OeBAS* and *CYP716C67*, which are highly expressed in the fruit, were functionally characterized and shown to catalyze key missing steps in the biosynthesis of olive fruit triterpenoids. Then, we confirmed the enzymatic functions of the entire pathway, by expressing the genes putatively involved in the synthesis of oleanane‐ and ursane‐type triterpenoids in a *N. benthamiana* platform (Reed *et al*., 2017). Our experiments show that *OeBAS* encodes an OSC required for β‐amyrin production and that *CYP716C67* is a C‐2α oxidase that oxygenates both the oleanane and ursane backbones to yield the 2α hydroxylated products maslinic and corosolic acid. The identification of this *CYP* is key for understanding the biosynthetic route to the major olive triterpenoids: oleanolic acid, maslinic acid, ursolic acid, uvaol, and erythrodiol. In fruit, the higher expression of *OeBAS*, which synthesizes the β‐amyrin skeleton of oleanane‐type triterpenoids, compared with *OeMAS* and *OeLUS*, might explain the higher accumulation of this type of triterpenoids in this organ (Stiti & Hartmann, 2012), compared with the ursane‐ and lupane‐type triterpenoids.

Our data also confirmed the function of *OeMAS* (Saimaru *et al*., 2007) and *CYP716A48* (Suzuki *et al*., 2018; Dale *et al*., 2020), and provided their genomic localization and expression profile in different organs. In accordance with Saimaru *et al*. (2007), we showed that *OeMAS* produces both α‐ and β‐amyrin from 2,3‐oxidosqualene, with a prevalence for the former product. Traces of ψ‐taraxasterol were also detected in *N. benthamiana* transiently expressing *OeMAS*. This compound was not detected in yeast possibly due to being below the limit of detection. OeMAS has also been reported to make small amounts of butyrospermol (Saimaru *et al*., 2007); however, we did not detect the presence of butyrospermol by OeMAS in either plant and yeast expression systems. Considering the relatively low amount of butyrospermol previously reported to be produced by OeMAS, our failure to detect this product might reflect detection limits, or it may coelute with other compounds in our GC–MS analysis.

In addition, we reported for the first time the activity of *OeMAS* in combination with the downstream CYP716s and showed that it provides the substrate for the following oxidations catalyzed by CYP716A48 and CYP716C67. The combinatorial activity of these three enzymes produced the ursane‐type triterpenoids uvaol, ursolic, and corosolic acid, and lower amount of oleanane‐type triterpenoids as erythrodiol and maslinic acid. We evaluated the activity of CYP716A48 toward both α‐ and β‐amyrin. This enzyme in presence of β‐amyrin produces oleanolic acid and minor amount of erythrodiol, according to its ability to catalyze two sequential oxidation reactions at C‐28. Similarly, it produces ursolic acid and uvaol in presence of α‐amyrin. It is previously reported that it is also able to oxidase lupeol for the synthesis of betulin and betulinic acid (Suzuki *et al*., 2018), as observed for the other C‐28 CYP716A involved in terpenoid biosynthesis in other plant species, able to oxidase oleanane, ursane and lupane backbones (Fukushima *et al*., 2011).

CYP716A is the largest and most common CYP716 subgroup in plants and its members commonly have C‐28 oxidizing activity toward pentacyclic triterpenoids (Fukushima *et al*., 2011). However, other CYP716A enzymes that oxidize different carbon positions have also been identified (Moses *et al*., 2015; Miettinen *et al*., 2017; Nakamura *et al*., 2018; Pütter *et al*., 2019).

In contrast to CYP716 A subgroup, the C subgroup, to which *CYP716C67* belongs, is not broadly distributed among plants. It is not yet known whether the members of this subgroup share the same functions, since that, up to now, only a few of them have been functionally characterized (Miettinen *et al*., 2017; Nakamura *et al*., 2018). Considering their spot distribution among plant orders, it is possible that the function of its members is not conserved and might rather reflect the specific evolution of plant clades (Miettinen *et al*., 2017). Among the characterized members, the *C. asiatica* CYP716C11 (Miettinen *et al*., 2017) and the *Avicennia marina* CYP716C53 (Nakamura *et al*., 2018) similarly to *O. europaea* CYP716C67 showed C‐2 hydroxylase activity on oleanolic acid and ursolic acid. CYP716C11 showed activity also on 6β‐hydroxyoleanolic acid and resulted specific for C‐28 carboxylated triterpenoids.

Analysis of the olive genome has allowed locating on chromosomes the new genes characterized in this study and characterized candidate triterpenoid biosynthetic genes. Most of these genes are unlinked, with the exception of *CYP716C67* and *OeMAS*, which are 1168 kb away from each other on chromosome 13 of cv Arbequina. However, the *OeMAS* position was not confirmed in the current version of the genome sequence of cv Leccino. Further analyses are needed to verify this issue.

A predicted triterpene biosynthetic gene cluster containing *OeLUS* was identified using plantiSMASH. This candidate cluster shares some features in common with a previously identified biosynthetic gene cluster in *Lotus japonicus* (the *AMY2* cluster; Krokida *et al*., 2013). However, it does not appear to have a role in triterpenoid biosynthesis in olive fruits. Further investigations are needed to investigate the role of this and other candidate biosynthetic gene clusters in olive.

The marker–trait association analysis allowed us to identify markers significantly linked to oleanolic and maslinic acid fruit content on chromosomes 15 and 13, where *OeBAS* and *CYP716C67* genes are located, respectively. As far as we know, these are the first reported trait‐associated markers for olive triterpenoids. The analysis of these chromosomes revealed also the presence of other candidate genes positioned upstream in the triterpenoid biosynthetic pathway. These results suggest that chromosomes 13 and 15 might play a relevant role in the control of olive triterpenoid content.

Further work is needed to identify genomic regions controlling fruit triterpenoid content by quantitative trait loci mapping, or studying the expression profile of target genes in the cross‐population, to verify any significant association between their expression and fruit triterpene content, as observed in apple (Andre *et al*., 2016). Most of the identified markers are distant from target biosynthesis genes; thus, their functional interactions with them need to be verified, considering that long‐distance chromatin interactions between loci might occur and chromatin loops might connect regulatory elements to their target genes (Peng *et al*., 2019). The analysis of the genomic regions surrounding the identified markers resulted in numerous TFs, some belonging to families known to be involved in the regulation of triterpenoid biosynthesis like bHLH, AP2‐like ethylene‐responsive and WRKY (Mertens *et al*., 2016; Tamura *et al*., 2018; da Silva Magedans *et al*., 2021; Suzuki *et al*., 2023). Further studies will assess their role in the regulation of olive triterpenoid biosynthesis.

As multifunctional bioactive compounds, triterpenoid high content in olive fruits and leaves is desirable and could be achieved through the selection of novel varieties by breeding. The identification of polymorphisms within gene sequences and markers linked to triterpene content should represent the most effective tools for the genomics‐assisted selection of high triterpenoid genotypes. Our work has allowed clarifying the pathway for the synthesis of main olive triterpenoids and provided genomic information that will drive all efforts aimed to increase triterpenoid compounds in olive leaves, fruits, and oil. These activities might be applied to the production of table olives, olive oil, and olive leaves with improved health‐beneficial properties.

## Competing interests

None declared.

## Author contributions

FA, AO and LB conceived the work. LB provided the plant samples and the sequence data. FA analyzed the transcriptomic data and searched for gene clusters in olive genome sequences. RT performed the phylogenetic trees. FA, OC, RT, NGMC sequenced and cloned the target genes. NGMC performed the cDNA synthesis and the RT‐qPCR experiments. FA performed the yeast expression experiments and the GC–MS analyses of yeast extracts. JR performed the *N. benthamiana* experiments and the GC–MS analyses of plant extracts. SM and RM collected the fruit samples from the cross‐population. AC and DT measured the oleanolic and maslinic acids content of the cross‐population. SM and RM performed the marker–trait association analysis. FA wrote the manuscript. All the authors critically revised the manuscript. FA and JR contributed equally to this work.

## Supporting information


**Fig. S1** Mass spectral data of oleanolic acid of olive fruits collected from Leccino × Dolce Agogia crossing population.
**Fig. S2** Mass spectral data of maslinic acid of olive fruits collected from Leccino × Dolce Agogia crossing population.
**Fig. S3** Calibration curves for the quantification of oleanolic and maslinic acids of olive fruits collected from Leccino × Dolce Agogia crossing population.
**Fig. S4** Relative mRNA levels of *OeBAS*, *OeMAS*, *CYP716A48*, and *CYP716C67* in olive organs.
**Fig. S5** GC–MS mass spectra of extracts from yeast cultures (*Saccharomyces cerevisiae*, GIL77 strain) expressing *OeBAS*, *OeMAS*, or empty vector.
**Fig. S6** Neighbor‐joining tree of olive CYP716s.
**Fig. S7** Hierarchical clustering of olive putative oxidosqualene cyclases and cytochromes P450 belonging to t716 family.
**Fig. S8** Alignment of CYP716A48 amino acid sequences of olive cv Leccino and cv Nevadillo Blanco.
**Fig. S9** Functional characterization of *OeBAS* and *OeMAS* in *Nicotiana benthamiana*.
**Fig. S10** Identification of ψ‐taraxasterol in *OeMAS*‐expressing *Nicotiana benthamiana* plants.
**Fig. S11** GC–MS mass spectra of extracts from *Nicotiana benthamiana* leaves expressing *OeBAS* with *tHMGR*, *CYP716A48v1*, and *CYP716C67*.
**Fig. S12** GC–MS mass spectra of extracts from *Nicotiana benthamiana* leaves expressing *OeMAS* with *tHMGR*, *CYP716A48v1*, and *CYP716C67*.
**Fig. S13** GC–MS mass spectra of extracts from *Nicotiana benthamiana* leaves expressing *OeBAS* or *OeMAS* with *tHMGR* and different cytochromes P450.
**Fig. S14** Lupeol synthase gene cluster in olive.
**Table S1** Co‐expression of olive *CYP716s* with *OeBAS*.
**Table S2** Primers used for reverse transcribed quantitative PCR analysis of olive genes and generation of constructs for heterologous expression in *Saccharomyces cerevisiae* and *Nicotiana benthamiana*.

**Table S3** Localization of oxidosqualene cyclases and *CYP716s* on olive genome.

**Table S4** Putative gene clusters for secondary metabolism in olive.

**Table S5** Segregant restriction associated DNA markers in olive genome significantly associated with oleanolic and maslinic acids content.
**Table S6** Localization of upstream genes of triterpenoid biosynthesis in olive chromosomes 13 and 15.

**Table S7** Transcription factors in the olive genomic regions surrounding the molecular markers of chromosomes 13 and 15.
Please note: Wiley is not responsible for the content or functionality of any Supporting Information supplied by the authors. Any queries (other than missing material) should be directed to the *New Phytologist* Central Office.

## Data Availability

Sequence data were submitted to the NCBI GenBank database under the following accession nos.: OP821086 (*OeBAS*v1), OP821087 (*OeBAS*v2), OP821088 (*CYP716A48v1*), OP821089 (*CYP716C67*v1), and OP821090 (*CYP716C67*v2). All the other data that support the findings of this study are available from the corresponding authors upon reasonable request.
