# Supplementary material for: OeBAS and CYP716C67 catalyze the biosynthesis of health‐beneficial triterpenoids in olive (Olea europaea) fruits
Source: New Phytol. 2023 Apr 3;238(5):2047–63. doi: 10.1111/nph.18863 (PMC10952584; doi:10.1111/nph.18863)
Supplement: Supplementary file 1 — Fig. S1 Mass spectral data of oleanolic acid of olive fruits collected from Leccino × Dolce Agogia crossing population. Fig. S2 Mass spectral data of maslinic acid of olive fruits collected from Leccino × Dolce Agogia crossing population. Fig. S3 Calibration curves for the quantification of oleanolic and maslinic acids of olive fruits collected from Leccino × Dolce Agogia crossing population. Fig. S4 Relative mRNA levels of OeBAS, OeMAS, CYP716A48, and CYP716C67 in olive organs. Fig. S5 GC–MS mass spectra of extracts from yeast cultures (Saccharomyces cerevisiae, GIL77 strain) expressing OeBAS, OeMAS, or empty vector. Fig. S6 Neighbor‐joining tree of olive CYP716s. Fig. S7 Hierarchical clustering of olive putative oxidosqualene cyclases and cytochromes P450 belonging to t716 family. Fig. S8 Alignment of CYP716A48 amino acid sequences of olive cv Leccino and cv Nevadillo Blanco. Fig. S9 Functional characterization of OeBAS and OeMAS in Nicotiana benthamiana. Fig. S10 Identification of ψ‐taraxasterol in OeMAS‐expressing Nicotiana benthamiana plants. Fig. S11 GC–MS mass spectra of extracts from Nicotiana benthamiana leaves expressing OeBAS with tHMGR, CYP716A48v1, and CYP716C67. Fig. S12 GC–MS mass spectra of extracts from Nicotiana benthamiana leaves expressing OeMAS with tHMGR, CYP716A48v1, and CYP716C67. Fig. S13 GC–MS mass spectra of extracts from Nicotiana benthamiana leaves expressing OeBAS or OeMAS with tHMGR and different cytochromes P450. Fig. S14 Lupeol synthase gene cluster in olive. Table S1 Co‐expression of olive CYP716s with OeBAS. Table S2 Primers used for reverse transcribed quantitative PCR analysis of olive genes and generation of constructs for heterologous expression in Saccharomyces cerevisiae and Nicotiana benthamiana. [file NPH-238-2047-s001.pdf]

***New Phytologist* Supporting Information**

Article title: *OeBAS* and *CYP716C67* catalyze the biosynthesis of health-beneficial triterpenoids in olive (*Olea europaea* L.) fruits

Authors: Fiammetta Alagna, James Reed, Ornella Calderini, Ramesha Thimmappa, Nicolò G.M. Cultrera, Alice Cattivelli, Davide Tagliazucchi, Soraya Mousavi, Roberto Mariotti, Anne Osbourn, Luciana Baldoni

Article acceptance date: 28 February 2023

The following Supporting Information is available for this article:

**Fig. S1** Mass spectral data of oleanolic acid of olive fruits collected from Leccino x Dolce Agogia crossing population.

**Fig. S2** Mass spectral data of maslinic acid of olive fruits collected from Leccino x Dolce Agogia crossing population.

**Fig. S3** Calibration curves for the quantification of oleanolic and maslinic acids of olive fruits collected from Leccino x Dolce Agogia crossing population.

**Fig. S4** Relative mRNA levels of *OeBAS*, *OeMAS*, *CYP716A48* and *CYP716C67* in olive organs.

**Fig. S5** GC-MS mass spectra of extracts from yeast cultures (*Saccharomyces cerevisiae*, GIL77 strain) expressing *OeBAS*, *OeMAS* or empty vector.

**Fig. S6** Neighbor-joining (Nj) tree of olive CYP716s.

**Fig. S7** Hierarchical clustering of olive putative oxidosqualene cyclases and cythocromes P450 belonging to 716 family.

**Fig. S8** Alignment of CYP716A48 amino acid sequences of olive cv. Leccino and cv. Nevadilo Blanco.

**Fig. S9** . Functional characterization of *OeBAS* and *OeMAS* in *Nicotiana benthamiana*.

**Fig. S10** Identification of ψ-taraxasterol in *OeMAS*-expressing *Nicotiana benthamiana* plants.

**Fig. S11** GC-MS mass spectra of extracts from *Nicotiana benthamiana* leaves expressing *OeBAS* with *tHMGR*, *CYP716A48v1* and *CYP716C67*.

**Fig. S12** GC-MS mass spectra of extracts from *Nicotiana benthamiana* leaves expressing *OeMAS* with *tHMGR*, *CYP716A48v1* and *CYP716C67*.

**Fig. S13** GC-MS mass spectra of extracts from *Nicotiana benthamiana* leaves expressing *OeBAS* or *OeMAS* with *tHMGR* and different cytochromes P450.

**Fig. S14** Lupeol synthase gene cluster in olive.

**Table S1** Coexpression of olive *CYP716s* with *OeBAS*.

**Table S2** Primers used for RT-qPCR analysis of olive and generation of constructs for heterologous expression in *Saccharomyces cerevisiae* and *Nicotiana benthamiana*.

**Table S3** Localization of *OSCs* and *CYP716s* on olive genome.

**Table S4** Putative gene clusters for secondary metabolism in olive.

**Table S5** Segregant restriction associated DNA (RAD) markers in olive genome significantly associated to oleanolic (OA) and maslinic (MA) acids content.

**Table S6** Localization of up-stream genes of triterpenoid biosynthesis in olive chromosomes 13 and 15.

**Table S7** Transcription factors in the olive genomic regions surrounding the molecular markers of chromosomes 13 and 15.

a

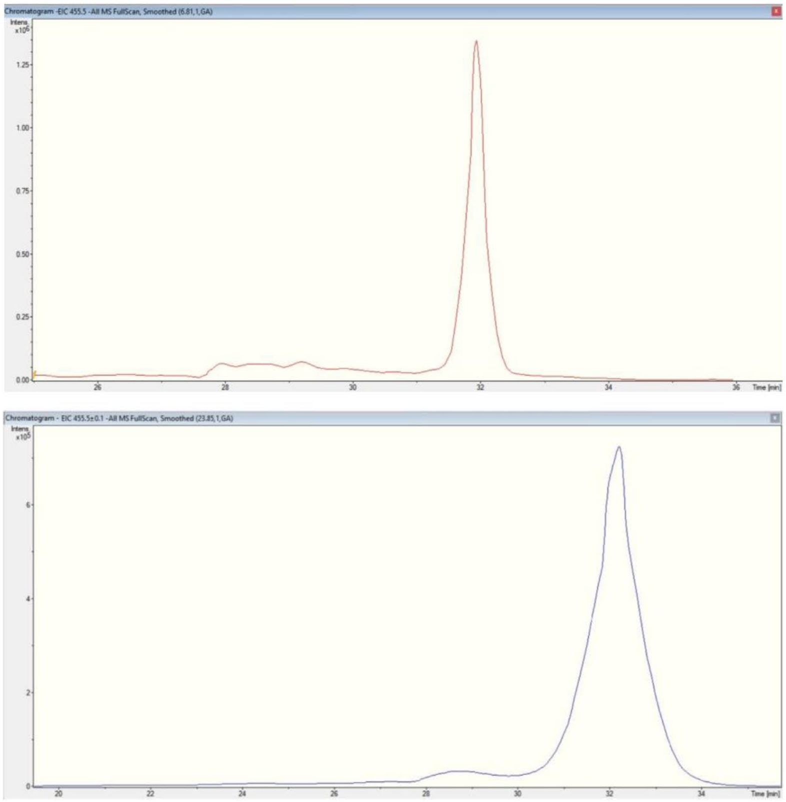

b

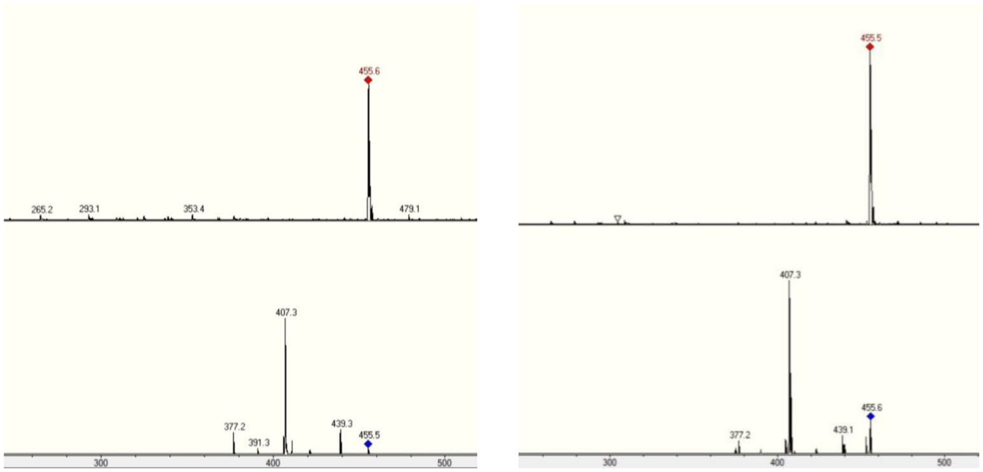


**Fig. S1** Mass spectral data of oleanolic acid of olive fruits collected from Leccino x Dolce Agogia crossing population. (a) Extracted ion chromatogram of the signal at m/z 455.5 (tolerance ± 0.5 Da) from a representative experiment (red), compared to the standard (blue). (b) Mass spectrum and MS/MS spectrum of oleanolic acid fragmentation. The figure shows the data

from a representative experiment (on the left) and from the standard (on the right).

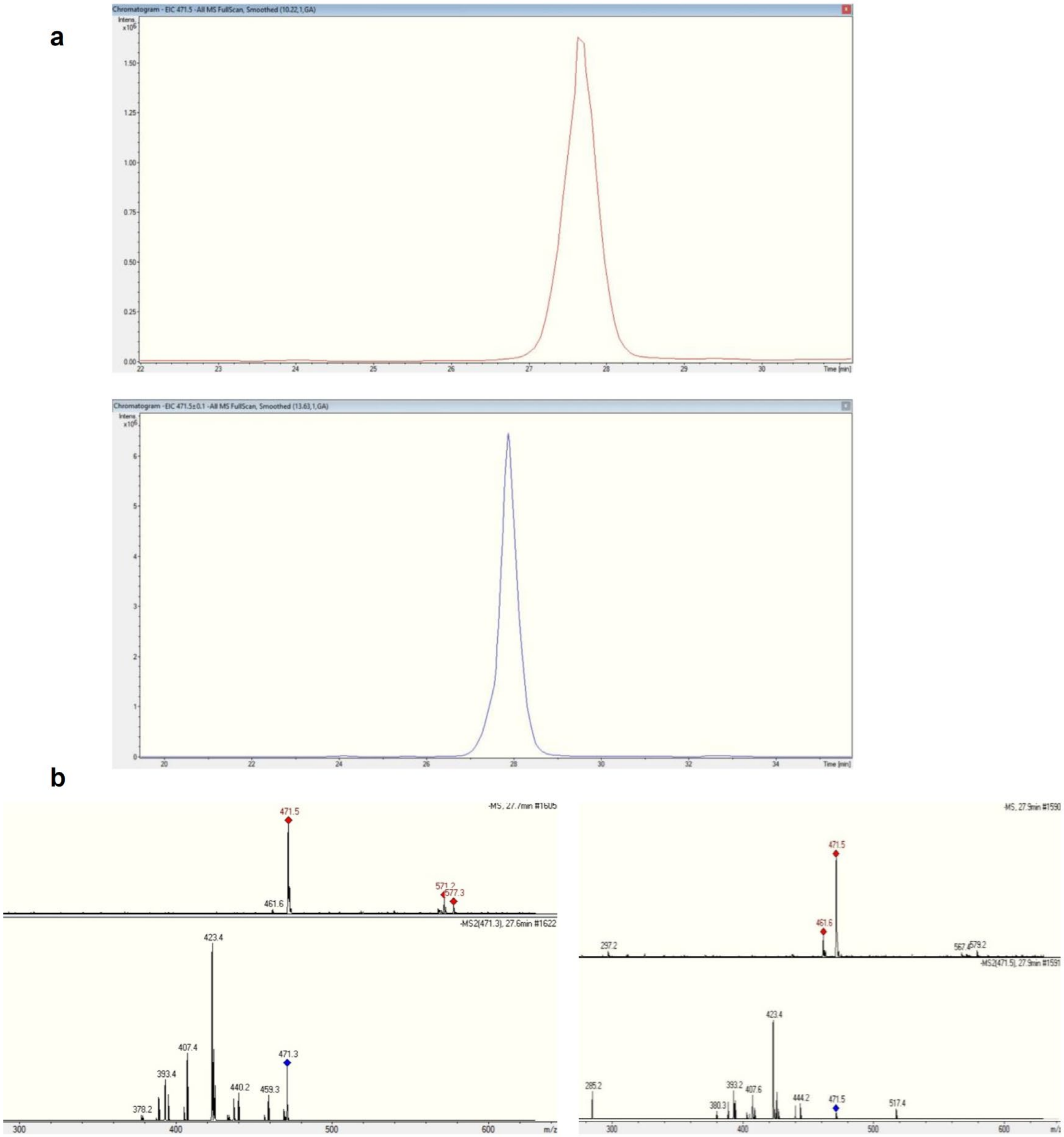


**Fig. S2** Mass spectral data of maslinic acid of olive fruits collected from Leccino x Dolce Agogia crossing population. (a) Extracted ion chromatogram of the signal at m/z 471.5 (tolerance ± 0.5 Da) from a representative experiment (red), compared to the standard (blue). (B) Mass spectrum and MS/MS spectrum of maslinic acid fragmentation. The figure shows the data from a representative experiment (on the left) and from the standard (on the right).

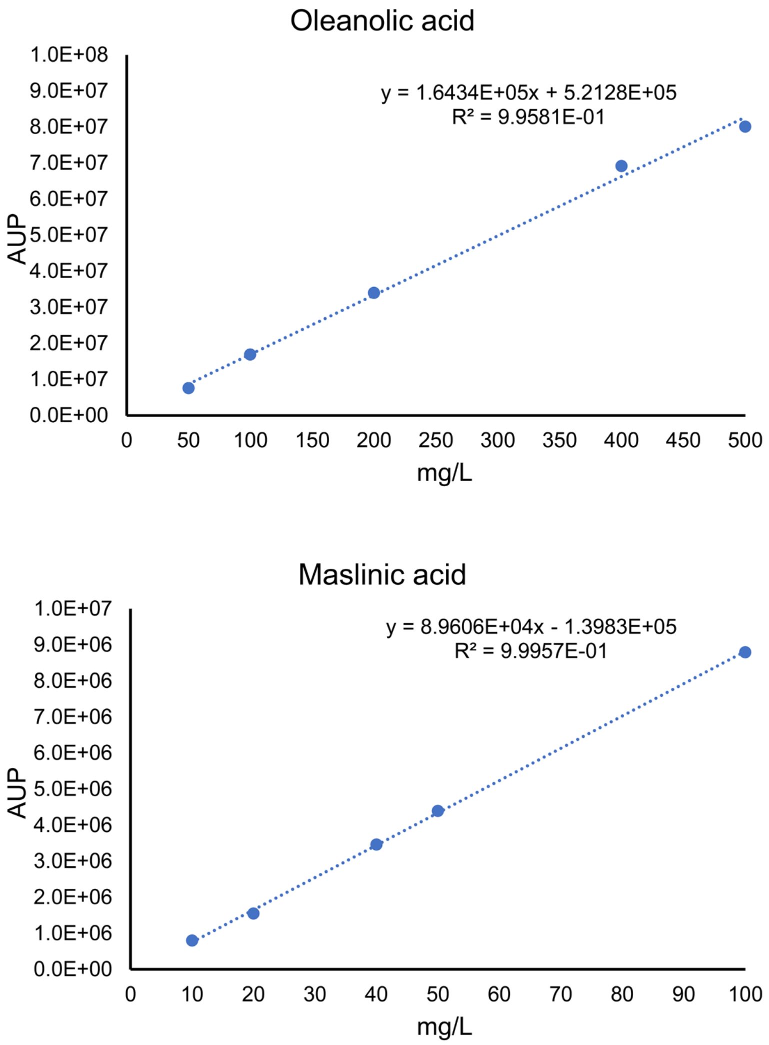


**Fig. S3** Calibration curves for the quantification of oleanolic and maslinic acids of olive fruits collected from Leccino x Dolce Agogia crossing population. The standards were dissolved in dimethyl sulfoxide (DMSO) and injected at different concentrations in the LC-MS system. The calibration curves were built by plotting the area under the peak (AUP), measured from the extracted ion chromatograms (tolerance ± 0.5 Da), for each injected standard solution, with the corresponding standard compound concentration.

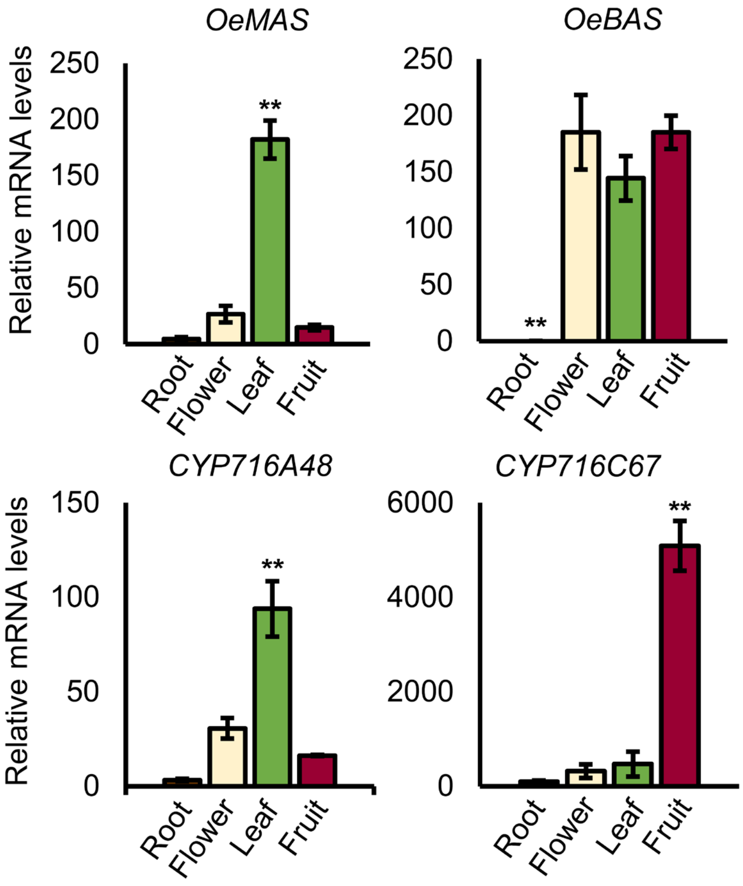


**Fig. S4** Relative mRNA levels of *OeBAS*, *OeMAS*, *CYP716A48* and *CYP716C67* in olive organs. The values are expressed as ΔΔCt. Bars, ± S.E. Statistically significant differences were determined by ANOVA followed by Tuckey's HSD post-hoc test on three biological replicates (** $p < 0.01$; n = 3).

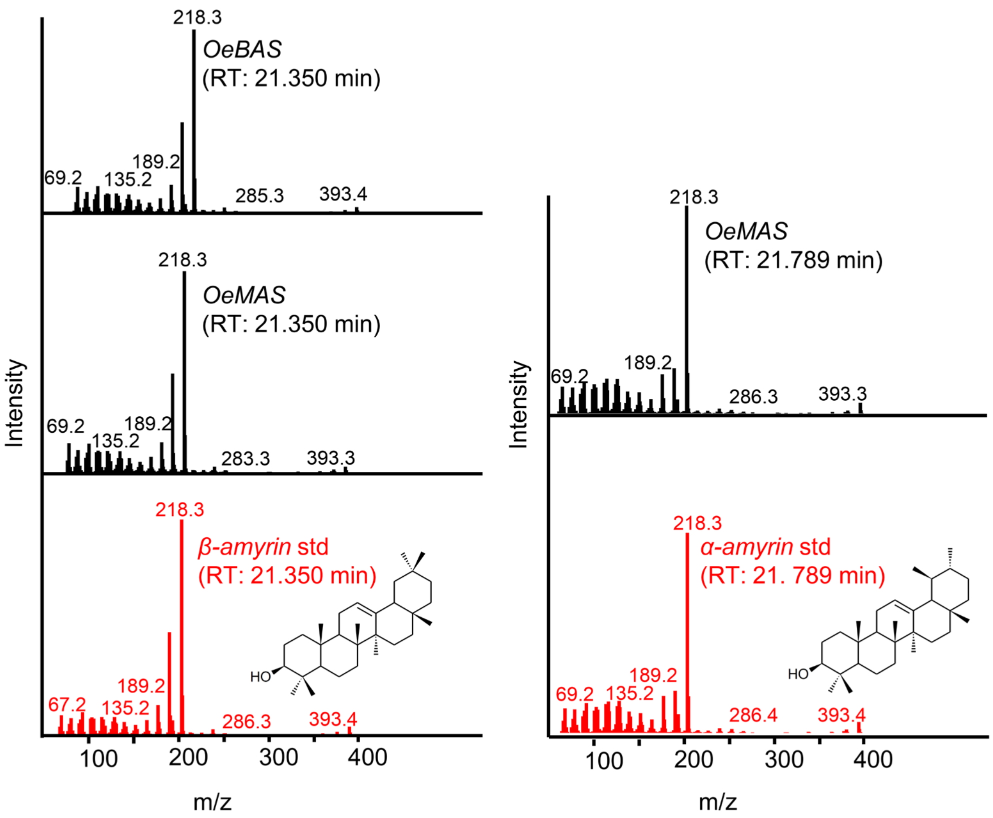


**Fig. S5** GC-MS mass spectra of extracts from yeast cultures (*Saccharomyces cerevisiae*, GIL77 strain) expressing *OeBAS*, *OeMAS* or empty vector. Data were compared with α-amyrin and β-amyrin standards (red indicated). RT: retention time.

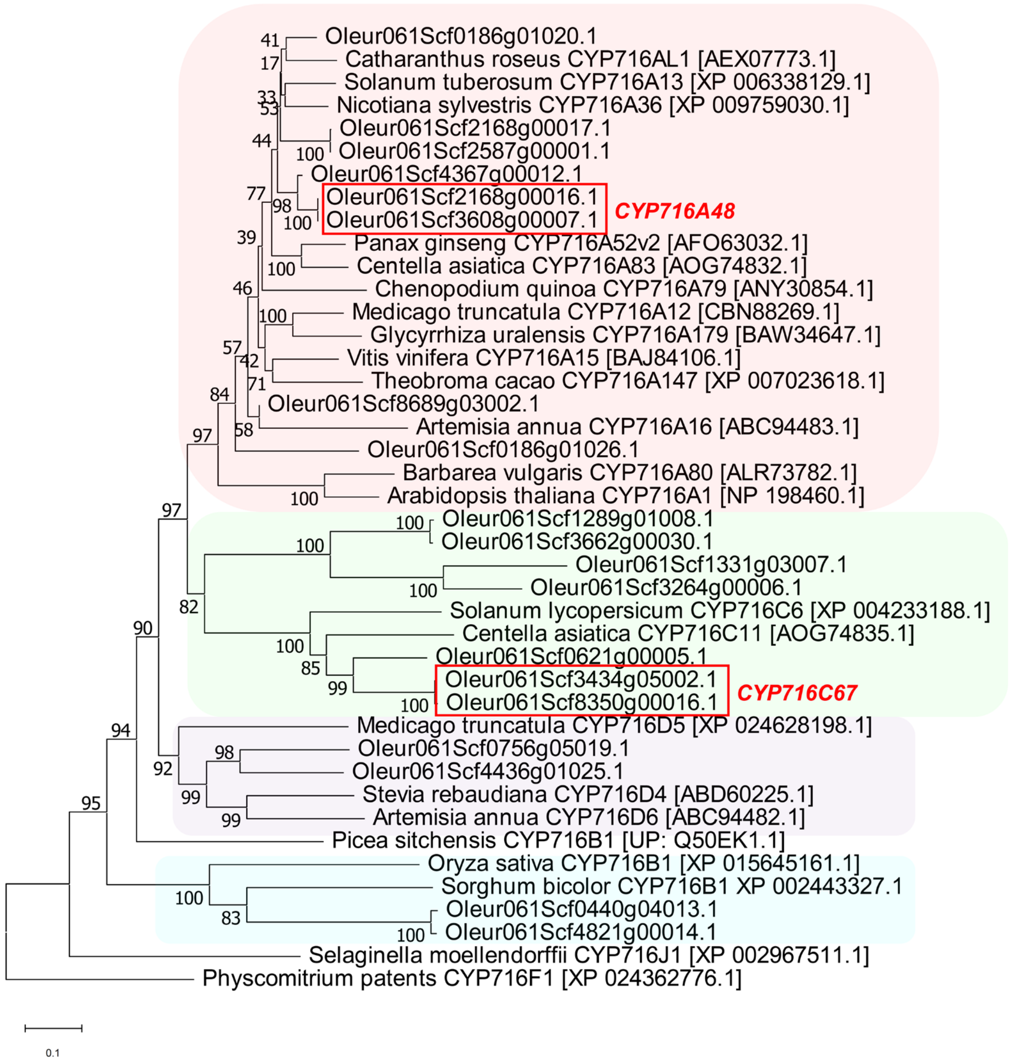


**Fig. S6** Neighbor-joining (Nj) tree of olive CYP716s. Amino acid sequences and classification were retrieved from the cytochrome P450 homepage (Nelson 2009). Nj tree was drawn using MEGA 10.2.6 as using parameters as shown in the methods. *Physcomitrium patens* (GenBankTM accession number XP_024362776.1) was used as outgroup. Bootstrap values are indicated at the branch nodes. Genbank accession numbers or UniProt ID (UP) are provided within brackets. Olive sequences were retrieved from olive genome sequencing data (Jiménez-Ruiz et al., 2020). Sequences selected for functional characterization in this study are indicated in red.

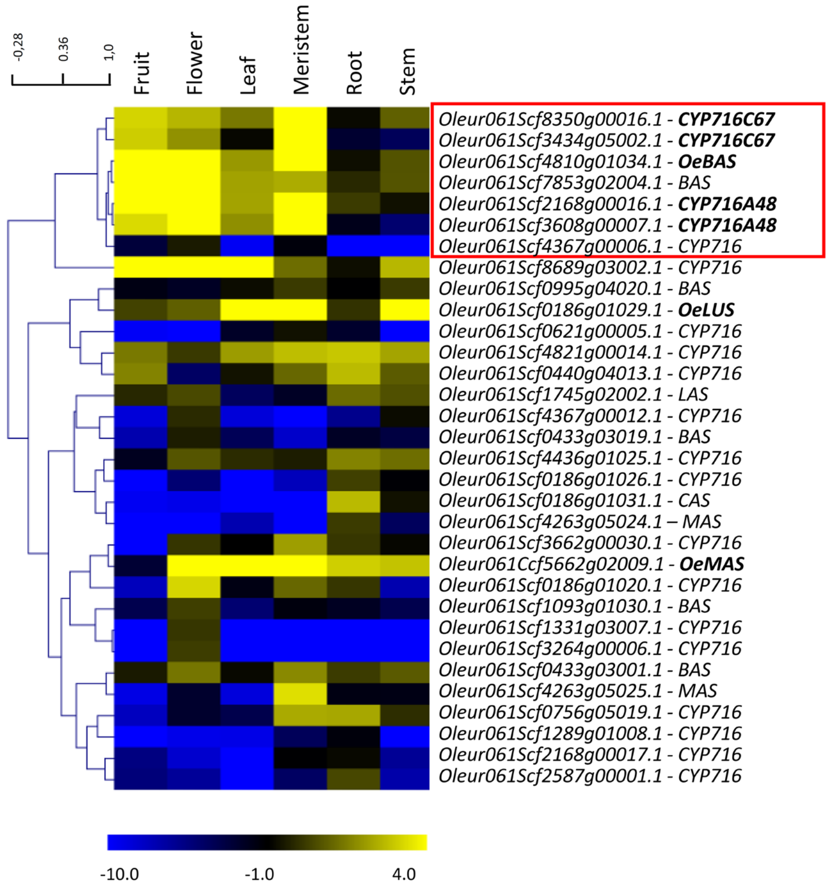


**Fig. S7** Hierarchical clustering of olive putative oxidosqualene cyclases and cythocromes P450 belonging to 716 family. For each gene, $Log_2$ RPKM was used for clustering analysis. Transcripts in bold correspond to those functionally characterized in this study. Genes were tentatively named based on their putative function (MAS: multifunctional α-amyrin synthase, CAS: cycloartenol synthase, BAS: β-amyrin synthase; LUS: lupeol synthase, LAS: lanosterol synthase; CYP716: cythocrome P450 belonging to 716 family). The expression values were retrieved by Ramírez-Tejero et al., 2020 and refers to transcripts mapped to cv. Picual genome (Jiménez-Ruiz et al., 2020). Sequences selected for functional characterization in this study are indicated in red. Oleur061Scf4367g00006.1 is a partial sequence of Oleur061Scf2168g00016.1/ Oleur061Scf3608g00007.1.

```
CYP716A48v1 cv Leccino       MEFFYVSLLCLFVLLISLSLHFLFYKNKSGFSGQIPPGKTGWPVIGESLEFLSNGWKGHP
CYP716A48v2 cv Nevadilo B.   MEFFYVSLLCLFVFLISLSLHFLFYKNKSSFSGQIPPGKTGWPVIGESLEFLSNGWKGHP
                             *************:***************.******************************

CYP716A48v1 cv Leccino       EKFIFDRIAKYSSYVFRTHLFGEPAAVFCGANGNKFLFSNENKLVQAWWPASVDKVFPSS
CYP716A48v2 cv Nevadilo B.   EKFIFDRIAKYSSYVFRTHLFGEPAAVFCGANGNKFLFSNENKLVQAWWPASVDKVFPSS
                             ************************************************************

CYP716A48v1 cv Leccino       NQTSSKEEAVKMRKMLPTFFKPEALQRYVGIMDHIAQRHFSDGWDNKNEVVVFPLAKRYT
CYP716A48v2 cv Nevadilo B.   NQTSSKEEAVKMRKMLPTFFKPEALQRYVGIMDHIAQRHFSDGWDNKNEVVVFPLAKRYT
                             ************************************************************

CYP716A48v1 cv Leccino       FWLACRLFVSVEDPAHVAKFADPFNELASGLISIPIDLPGTPFHRAIKSSNFIRKELVSI
CYP716A48v2 cv Nevadilo B.   FWLACRLFVSVEDPAHVAKFADPFNELASGLISIPIDLPGTPFHRAIKSSNFIRKELVSI
                             ************************************************************

CYP716A48v1 cv Leccino       IKQRKIDLAEGKASPTQDILSHMLLTSDESGKFMHELDIADKILGLLVGGHDTASSACTF
CYP716A48v2 cv Nevadilo B.   IKQRKIDLAEGKASPTQDILSHMLLTSDESGKFMHELDIADKILGLLVGGHDTASSACTF
                             ************************************************************

CYP716A48v1 cv Leccino       VVKYLAELPEIYEGVYQEQMEIAKSKAPGELLNWDDIQKMKYSWNVACEVLRLAPPLQGA
CYP716A48v2 cv Nevadilo B.   VVKYLAELPEIYEGVYQEQMEIAKSKAPGELLNWDDIQKMKYSWNVACEVLRLAPPLQGA
                             ************************************************************

CYP716A48v1 cv Leccino       FREAITDFMFNGFSIPKGWKLYWSANSTHRNSEFFPEPLKFDPSRFEGSGPAPYTFVPFG
CYP716A48v2 cv Nevadilo B.   FREAITDFMFNGFSIPKGWKLYWSANSTHRNSEFFPEPLKFDPSRFEGSGPAPYTFVPFG
                             ************************************************************

CYP716A48v1 cv Leccino       GGPRMCPGKEYARLEILVFMHHLVKRFKWEKLIPDEKIVVDPMPIPAKGLPIRLYPHNA
CYP716A48v2 cv Nevadilo B.   GGPRMCPGKEYARLEILVFMHHLVKRFKWEKLIPDEKIVVDPMPIPAKGLPIRLYPLNA
                             ******************************************************** **
```

**Fig. S8** Alignment of CYP716A48 amino acid sequences of olive cv. Leccino and cv. Nevadilo Blanco. The CYP716A48 protein retrieved from cv. Leccino (CYP716A48v1) has been functionally characterized in this study, whereas, cv. Nevadilo Blanco’s protein (CYP716A48v2) was previously characterized by Suzuki et al., 2018. The alignment was performed by MUSCLE Multiple Sequence Alignment (https://www.ebi.ac.uk/Tools/msa/muscle/).

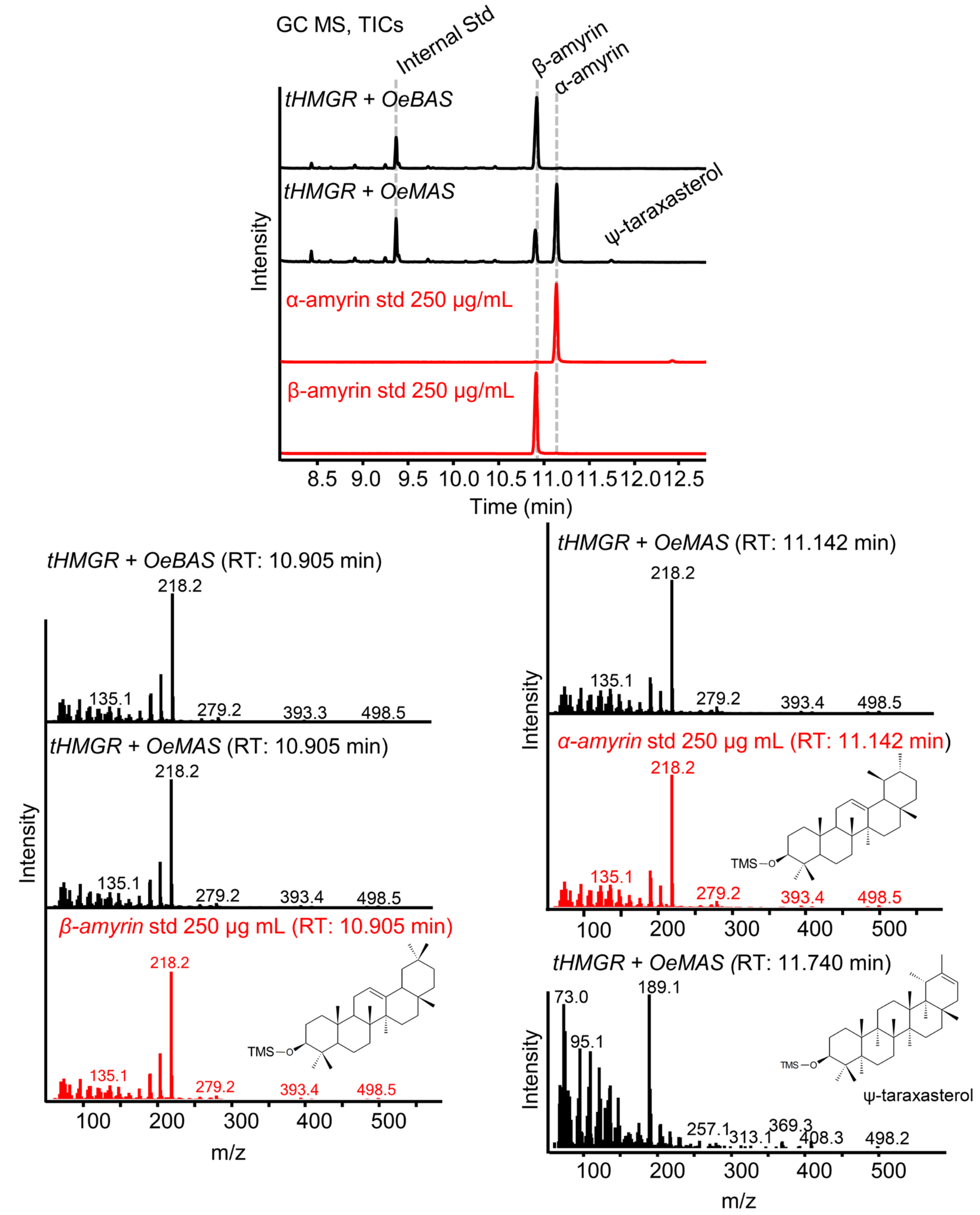


**Fig. S9** . Functional characterization of *OeBAS* and *OeMAS* in *Nicotiana benthamiana*. GC-MS total ion chromatograms (TICs) and related MS spectra for extracts from *N. benthamiana* leaves expressing *OeBAS* or *OeMAS* with *tHMGR* (Reed et al., 2017) compared with 250μg/mL of α-amyrin and β-amyrin standards (red indicated). OeMAS is the mixed α-amyrin synthase identified by Saimaru and coauthors (2007). RT: retention time.

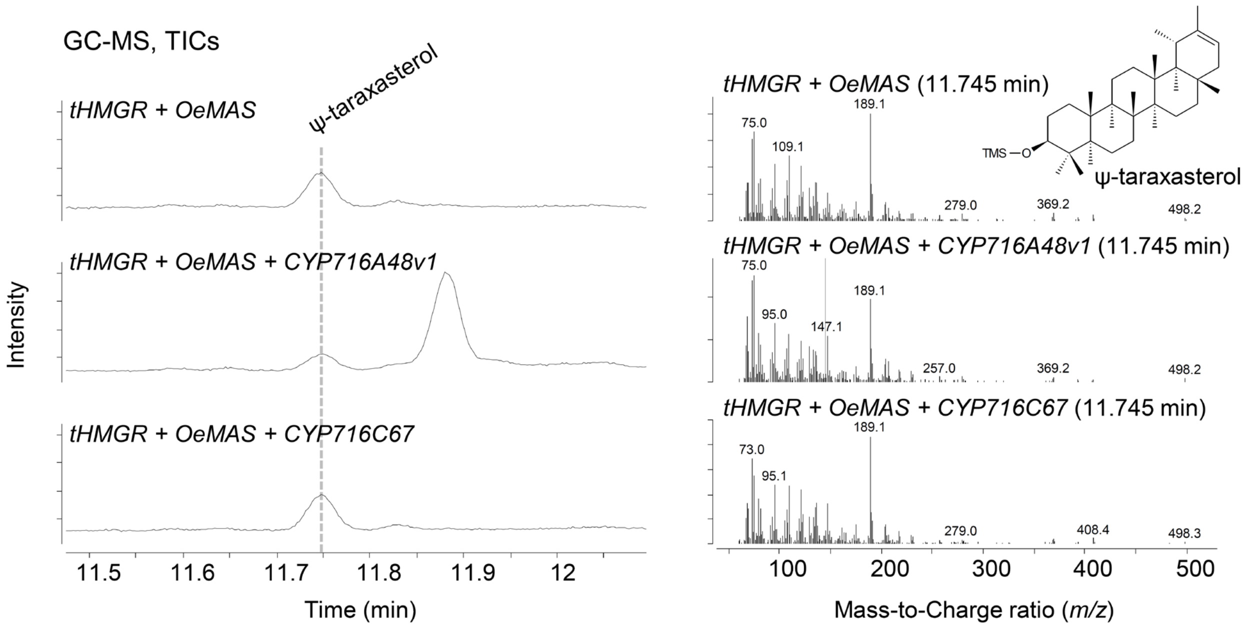


**Fig. S10** Identification of ψ-taraxasterol in *OeMAS*-expressing *Nicotiana benthamiana* plants. GC-MS total ion chromatograms (TICs) and related MS spectra for extracts from *N. benthamiana* leaves expressing *OeMAS* with *tHMGR*, (Reed et al., 2017), *CYP716A48v1* and *CYP716C67*. OeMAS is the mixed α-amyrin synthase identified by Saimaru and coauthors (2007).

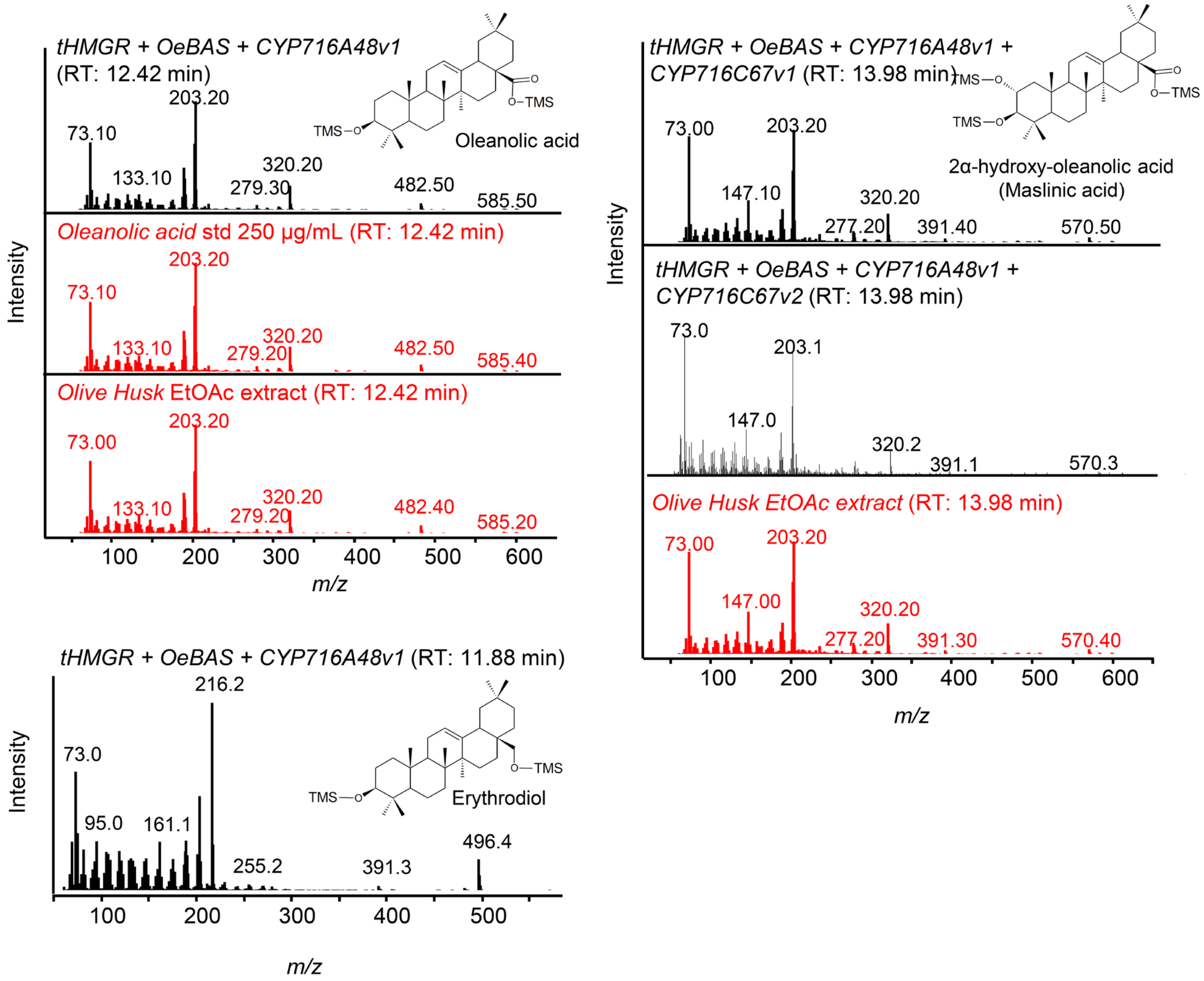


**Fig. S11** GC-MS mass spectra of extracts from *Nicotiana benthamiana* leaves expressing *OeBAS* with *tHMGR*, *CYP716A48v1* and *CYP716C67*. Mass spectra were compared with olive husk ethyl acetate extract and 250µg/mL of oleanolic acid standard; RT: retention time.

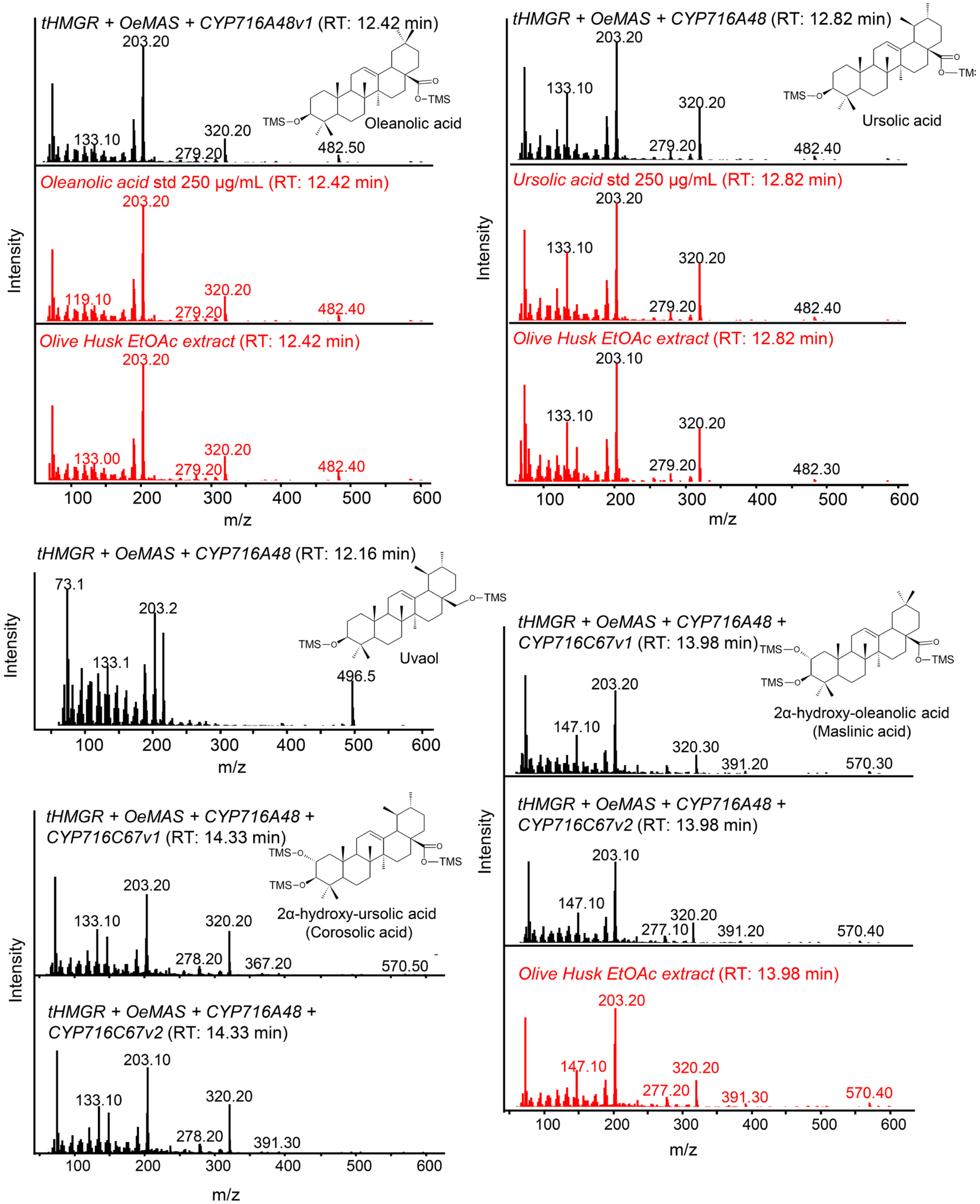


**Fig. S12** GC-MS mass spectra of extracts from *Nicotiana benthamiana* leaves expressing *OeMAS*

with *tHMGR*, *CYP716A48v1* and *CYP716C67*. Mass spectra were compared with olive husk ethyl acetate extract, 250µg/mL of oleanolic acid or ursolic acid standards; RT: retention time.

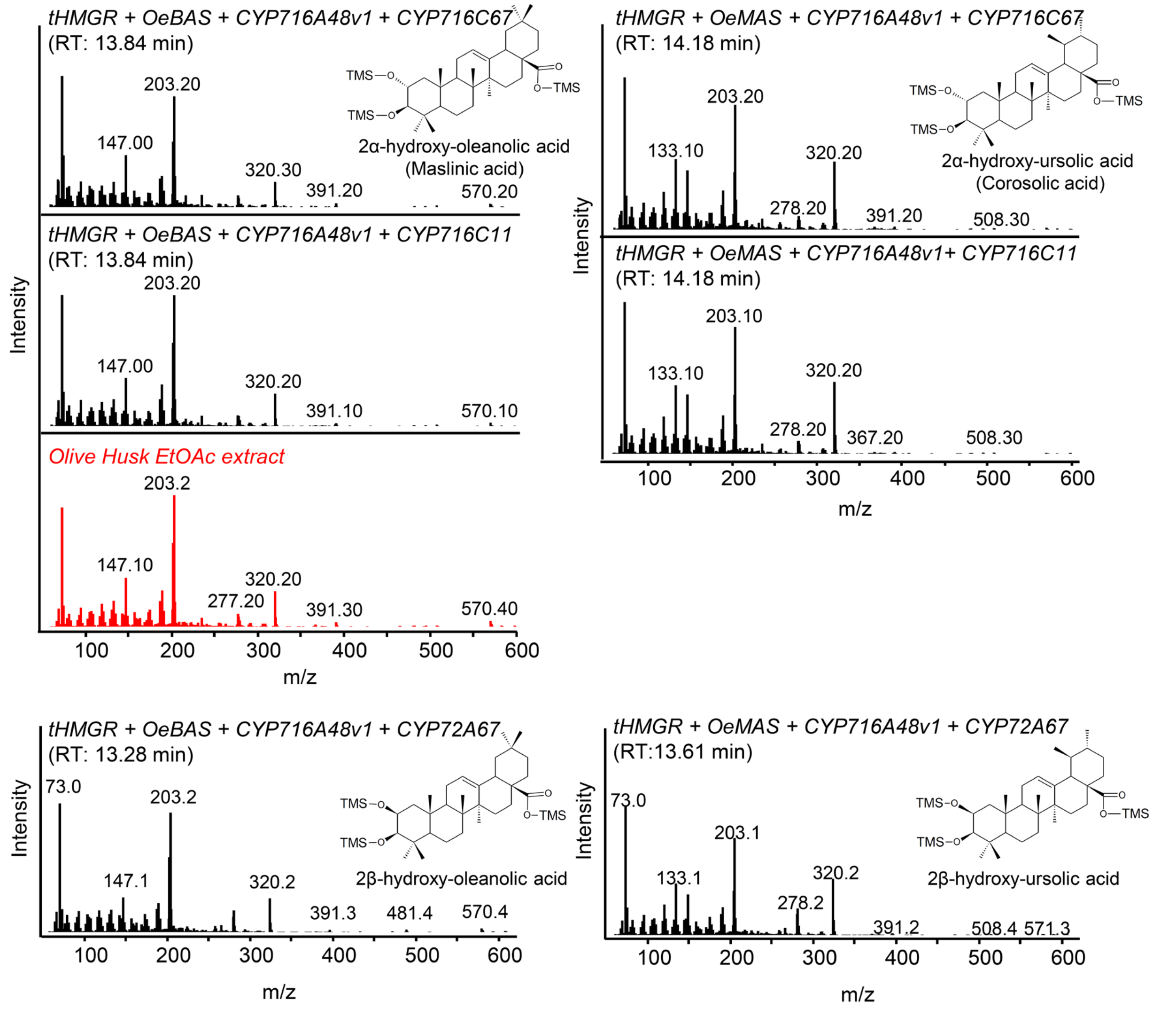


**Fig. S13** GC-MS mass spectra of extracts from *Nicotiana benthamiana* leaves expressing *OeBAS* or *OeMAS* with *tHMGR* and different cytochromes P450. *OeBAS* or *OeMAS* were co-expressed with *tHMGR*, *CYP716A48v1* and *CYP716C67*. For comparison, *OeBAS* and *CYP716A48v1* were co-expressed with either *Centella asiatica CYP716C11* (oleanolic acid 2α hydroxylase) or *Medicago truncatula CYP72A67* (oleanolic acid 2β hydroxylase). Similarly, *OeMAS* was co-expressed with

the same genes. RT: retention time.

**a**

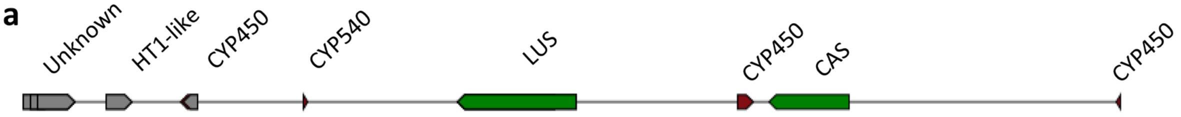


| Gene | Gene ID | Annotation | From | To | Strand |
|---|---|---|---|---|---|
| Unknown | OE9A002075<br>OE9A0020 | Unknown | 5965664<br>5965664 | 5969595<br>5972682 | + |
| HT1-like | OE9A012916 | Serine/Threonine-protein kinase HT1-like | 5977084 | 5980574 | + |
| CYP450_1 | OE9A051613 | Cytochrome P450 | 5987142 | 5989535 | - |
| CYP450_2 | OE9A116188 | Cytochrome P450 | 6003884 | 6004471 | + |
| LUS | OE9A083707 | Lupeol synthase | 6024705 | 6040977 | - |
| CYP450_3 | OE9A087787 | Cytochrome P450 | 6062876 | 6064969 | + |
| CAS | OE9A040168 | Cycloartenol synthase | 6067030 | 6078007 | - |
| CYP450_4 | OE9A007288 | Cytochrome P450 | 6114247 | 6114754 | - |

**b**

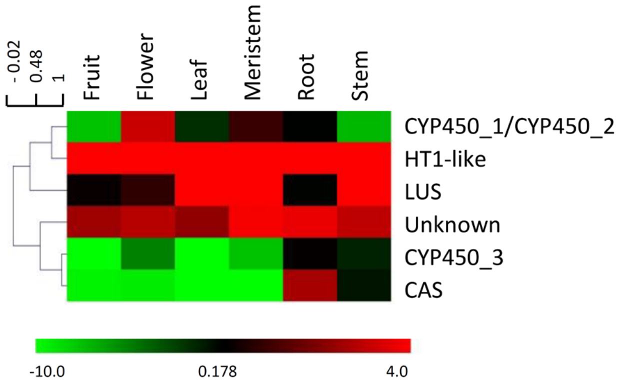


**Fig. S14** Lupeol synthase gene cluster in olive. (a) A schematic representation of triterpene gene cluster (Gene Cluster 16), as identified by plantiSMASH web tool (Kautsar et al., 2017) uploading olive genome sequences (cv. Farga, OE9 assembly) and annotations (Cruz et al., 2016). (b) Hierarchical clustering of target genes. For each gene, $Log_2$ RPKM was used for clustering analysis. The expression values were retrieved by Ramírez-Tejero et al., 2020 and refers cv. Picual transcripts (Jiménez-Ruiz et al., 2020) identified by BLASTn using the above genes as query. The expression of the best hits was considered. The following transcripts were

considered for each gene: CYP450_1 = Oleur061Scf0186g01020.1, CYP450_2 = Oleur061Scf0186g01020.1, HT1-like = Oleur061Scf0186g01024.1, Unknown = Oleur061Scf0186g00005.1; CYP450_3 = Oleur061Scf0186g01026.1, CAS = Oleur061Scf0186g01031.1. Expression data for CYP450_4 were not available.

**Table S1** Coexpression of olive *CYP716s* with *OeBAS*. Pearson correlation coefficient is reported. The genes indicated in red has been selected for characterization studies for their high P value and high expression in olive fruits.

| Gene ID | P |
|---|---|
| Oleur061Scf8350g00016.1 | 0.711518 |
| Oleur061Scf3434g05002.1 | 0.777518 |
| Oleur061Scf2168g00016.1 | 0.970434 |
| Oleur061Scf3608g00007.1 | 0.912802 |
| Oleur061Scf8689g03002.1 | 0.444426 |
| Oleur061Scf0621g00005.1 | -0.37932 |
| Oleur061Scf4821g00014.1 | -0.79595 |
| Oleur061Scf0440g04013.1 | -0.7343 |
| Oleur061Scf4367g00012.1 | 0.069521 |
| Oleur061Scf4436g01025.1 | -0.46889 |
| Oleur061Scf0186g01026.1 | -0.49884 |
| Oleur061Scf3662g00030.1 | -0.143 |
| Oleur061Scf0186g01020.1 | 0.347186 |
| Oleur061Scf1331g03007.1 | 0.744821 |
| Oleur061Scf3264g00006.1 | 0.720294 |
| Oleur061Scf0756g05019.1 | -0.41913 |
| Oleur061Scf1289g01008.1 | -0.41119 |
| Oleur061Scf2168g00017.1 | -0.28504 |
| Oleur061Scf2587g00001.1 | -0.28841 |

**Table S2** Primers used for RT-qPCR analysis of olive genes and generation of constructs for

heterologous expression in *Saccharomyces cerevisiae* and *Nicotiana benthamiana*. Where Gateway *attB* sites were included in primers, these sequences are underlined.

| Application | Primer | Target | Sequence (5'-3') |
|---|---|---|---|
| RT-qPCR | P1-fw | *OeMAS* | GAAGGAAGACAATGGCCCGA |
| | P1-rev | *OeMAS* | CAGCAGACAATCCTCCAAGCA |
| | P2-fw | *OeBAS* | ATCCCCAACAGGAAATCACA |
| | P2-rev | *OeBAS* | GCTAGAGCCCACAATGGGTA |
| | P3-for | *CYP716A48* | CAATGCCGATTCCTGCTAAG |
| | P3-rev | *CYP716A48* | AGGGCCAAATTGGTCACACT |
| | P4-for | *CYP716C67* | GCCTTTAGTTGGGTGCATGT |
| | P4-rev | *CYP716C67* | ATCCCCTCTTCTCATTACATCTTTC |
| Generation of constructs for heterologous expression | P5-for | *OeBAS* | ATGTGGAAATTGAAGATTGCAGAAGG |
| | P5-rev | *OeBAS* | TTAAATCTTTTTGGATGGTAATAGAACC |
| | P6-for | *OeMAS* | ATGTGGAAGCTTAAGATTGCTGAAGGA |
| | P6-rev | *OeMAS* | TTACAGGCTTTGAGATGACCACACAC |
| | P7-for | *CYP716A48* | ATGGAGTTCTTCTATGTCTCTCTTC |
| | P7-rev | *CYP716A48* | TTAAGCATTATGGGGATARAGACGAA |
| | P8-for | *CYP716C67* | GGGGACAAGTTTGTACAAAAAAGCAGGCTTCATGGAAGTTTTTGCTCTAGCCCTT |
| | P8-rev | *CYP716C67* | GGGGACCACTTTGTACAAGAAAGCTGGGTCCTAAAGGCGATGAAGGCGGATTG |
| | P9-for | *CYP72A67* | GGGGACAAGTTTGTACAAAAAAGCAGGCTTAATGGAAGCATCATTGGCC |
| | P9-rev | *CYP72A67* | GGGGACCACTTTGTACAAGAAAGCTGGGTATTATGCTTTCACTTTGCGTAG |

**Table S3** Localization of *OSCs* and *CYP716s* on olive genome. Position of candidate genes in olive genome of cv. Leccino, v3 assembly (http://olgenome.crea.gov.it/) and cv. Arbequina (Rao et al., 2021). Gene ID of best hits are reported, as assessed by BLAST.

**Table S4** Putative gene clusters for secondary metabolism in olive. Gene clusters were identified by plantiSMASH web tool (Kautsar et al., 2017) uploading olive genome sequences (cv. Farga, OE9 assembly) and annotations (Cruz et al., 2016). Lupeol synthase gene cluster is highlighted.

**Table S5** Segregant restriction associated DNA (RAD) markers in olive genome significantly associated to oleanolic (OA) and maslinic (MA) acids content.

**Table S6** Localization of up-stream genes of triterpenoid biosynthesis in olive chromosomes 13 and 15. Position of candidate genes in olive genome of cv. Leccino, v3 assembly (http://olgenome.crea.gov.it/) are shown.

| Gene name | Gene ID | Putative Function | Chr | Position (Kbp) |
|---|---|---|---|---|
| ***OeMVAK*** | Oe15g182490.t01 | Mevalonate kinase (EC:2.7.1.36) | Chr 15 | 40,581 |
| ***OeMVAK*** | Oe15g706810.t01 | Mevalonate kinase (EC:2.7.1.36) | Chr 15 | 41,248 |
| ***OeMVAPK*** | Oe13g508390.t02 | Phosphomevalonate kinase (EC:2.7.4.2) | Chr 13 | 23,319 |
| ***OeMVAPPD*** | Oe13g231070.t01 | Mevalonate diphosphate decarboxylase (EC:4.1.1.33) | Chr 13 | 31,119 |
| ***OeSQS*** | Oe15g538060.t01 | Squalene synthase (EC: 2.5.1.21) | Chr 15 | 12,574 |
| ***OeSQS*** | Oe15g150160.t01 | Squalene synthase (EC: 2.5.1.21) | Chr 15 | 39,044 |

**Table S7** Transcription factors in the olive genomic regions surrounding the molecular markers of chromosomes 13 and 15.

## References

**Cruz F, Julca I, Gómez-Garrido J, Loska D, Marcet-Houben M, Cano E, Galán B, Frias L, Ribeca P, Derdak S, *et al.* 2016**. Genome sequence of the olive tree, Olea europaea. *GigaScience* **5**: s13742-016.

**Jiménez-Ruiz J, Ramírez-Tejero JA, Fernández-Pozo N, Leyva-Pérez M de la O, Yan H, Rosa R de la, Belaj A, Montes E, Rodríguez-Ariza MO, Navarro F, *et al.* 2020**. Transposon activation is a major driver in the genome evolution of cultivated olive trees (Olea europaea L.). *Plant Genome* **13**: 1–19.

**Kautsar SA, Suarez Duran HG, Blin K, Osbourn A, Medema MH**. **2017**. plantiSMASH: automated identification, annotation and expression analysis of plant biosynthetic gene clusters. *Nucleic Acids Research* **45**: W55–W63.

**Mariotti R, Fornasiero A, Mousavi S, Cultrera NGM, Brizioli F, Pandolfi S, Passeri V, Rossi M, Magris G, Scalabrin S, *et al.* 2020**. Genetic Mapping of the Incompatibility Locus in Olive and Development of a Linked Sequence-Tagged Site Marker. *Frontiers in Plant Science* **10**: 1760.

**Ramírez-Tejero JA, Jiménez-Ruiz J, de la O Leyva-Pérez M, Barroso JB, Luque F**. **2020**. Gene Expression Pattern in Olive Tree Organs. *Genes* **11**: 544.

**Rao G, Zhang J, Liu X, Lin C, Xin H, Xue L, Wang C**. **2021**. De novo assembly of a new Olea europaea genome accession using nanopore sequencing. *Horticulture Research* **8**: 64.

**Reed J, Stephenson MJ, Miettinen K, Brouwer B, Leveau A, Brett P, Goss RJM, Goossens A, O'Connell MA, Osbourn A**. **2017**. A translational synthetic biology platform for rapid access to gram-scale quantities of novel drug-like molecules. *Metabolic Engineering* **42**: 185–193.

**Saimaru H, Orihara Y, Tansakul P, Kang Y-H, Shibuya M, Ebizuka Y**. **2007**. Production of Triterpene Acids by Cell-suspension Cultures of Olea europaea. *Chem. Pharm. Bull* **55**: 784–788.

**Suzuki H, Fukushima EO, Umemoto N, Ohyama K, Seki H, Muranaka T**. **2018**. Comparative analysis of CYP716A subfamily enzymes for the heterologous production of C-28 oxidized triterpenoids in transgenic yeast. *Plant Biotechnology* **35**: 131–139.
